# High-resolution transcriptional and morphogenetic profiling of cells from micropatterned human ESC gastruloid cultures

**DOI:** 10.7554/eLife.59445

**Published:** 2020-11-18

**Authors:** Kyaw Thu Minn, Yuheng C Fu, Shenghua He, Sabine Dietmann, Steven C George, Mark A Anastasio, Samantha A Morris, Lilianna Solnica-Krezel

**Affiliations:** 1 Department of Biomedical Engineering, Washington UniversitySt. LouisUnited States; 2 Department of Developmental Biology, Washington University School of MedicineSt. LouisUnited States; 3 Department of Genetics, Washington University School of MedicineSt. LouisUnited States; 4 Center of Regenerative Medicine, Washington University School of MedicineSt. LouisUnited States; 5 Department of Computer Science & Engineering, Washington UniversitySt. LouisUnited States; 6 Division of Nephrology, Washington University School of MedicineSt. LouisUnited States; 7 Institute for Informatics, Washington University School of MedicineSt. LouisUnited States; 8 Department of Biomedical Engineering, University of California, DavisDavisUnited States; 9 Department of Bioengineering, University of IllinoisUrbana-ChampaignUnited States; University of Wisconsin, MadisonUnited States; University of California, BerkeleyUnited States

**Keywords:** gastrulation, germ layers, cell sorting, primordial germ cells, amnion, trophectoderm, Human

## Abstract

During mammalian gastrulation, germ layers arise and are shaped into the body plan while extraembryonic layers sustain the embryo. Human embryonic stem cells, cultured with BMP4 on extracellular matrix micro-discs, reproducibly differentiate into gastruloids, expressing markers of germ layers and extraembryonic cells in radial arrangement. Using single-cell RNA sequencing and cross-species comparisons with mouse, cynomolgus monkey gastrulae, and post-implantation human embryos, we reveal that gastruloids contain cells transcriptionally similar to epiblast, ectoderm, mesoderm, endoderm, primordial germ cells, trophectoderm, and amnion. Upon gastruloid dissociation, single cells reseeded onto micro-discs were motile and aggregated with the same but segregated from distinct cell types. Ectodermal cells segregated from endodermal and extraembryonic but mixed with mesodermal cells. Our work demonstrates that the gastruloid system models primate-specific features of embryogenesis, and that gastruloid cells exhibit evolutionarily conserved sorting behaviors. This work generates a resource for transcriptomes of human extraembryonic and embryonic germ layers differentiated in a stereotyped arrangement.

## Introduction

During mammalian embryogenesis, the first major lineage segregation occurs when trophectoderm (TE), hypoblast (primitive endoderm), and pluripotent epiblast arise in the blastocyst (Cockburn and Rossant, 2010). Epiblast is a precursor of all embryonic tissues, while the surrounding hypoblast and TE form extraembryonic (ExE) cells that provide signals to instruct embryonic polarity and germ layers formation (Lawson et al., 1991; Arnold and Robertson, 2009). Despite similarities at the pre-implantation blastocyst stage, differences are observed during post-implantation stages between primate and murine development, as revealed by in vitro cultured human (Deglincerti et al., 2016; Shahbazi et al., 2016) and cynomolgus (cyno) monkey embryos (Ma et al., 2019; Nakamura et al., 2016; Niu et al., 2019). Whereas TE is further distinguished into polar (adjacent to epiblast) and mural (distal to epiblast) in both murine and primate preimplantation embryos, implantation is initiated via polar TE in primates but via mural TE in murine embryos (Shahbazi and Zernicka-Goetz, 2018). Upon implantation in human and primate cyno monkey embryos, epiblast cells adjacent to the polar TE form amnion, while those adjacent to hypoblast form a flat epithelial disc that will give rise to germ layers during gastrulation (Ma et al., 2019; Niu et al., 2019; Xiang et al., 2020). In contrast, germ layers arise from cup-shaped epiblast epithelium in mouse.

Gastrulation, during which germ layers are formed and shaped into a body plan, is a fundamental and conserved phase of animal embryogenesis (Solnica-Krezel and Sepich, 2012). During mouse and cyno monkey gastrulation, the primitive streak (PS) forms at the posterior epithelial epiblast. Cells in this region undergo epithelial to mesenchymal transition (EMT) and subsequently ingress through the PS and migrate to form mesoderm and endoderm. Cells that remain in the epiblast become ectoderm (Tam and Behringer, 1997; Williams et al., 2012). Concurrent with gastrulation initiation, primordial germ cells (PGCs) are specified in the epiblast of the mouse embryo (Lawson et al., 1999), but likely in the amnion in primates (Sasaki et al., 2016).

While EMT endows mesendodermal cells with motility, another morphogenetic process known as cell sorting is thought to underlie the proper segregation of germ layers in frog and fish gastrulae. Through cell sorting, germ layers achieve and maintain segregated populations, ensuring the establishment of tissue boundaries (Fagotto, 2014). First demonstrated by Holtfreter and colleagues, cells from dissociated amphibian gastrulae, when re-aggregated in vitro, segregate into their distinct germ layers (Townes and Holtfreter, 1955). Subsequent work in *Xenopus*, *Rana pipiens*, and zebrafish gastrulae further supports reaggregation and segregation of germ layer cells (Davis et al., 1997; Klopper et al., 2010; Ninomiya and Winklbauer, 2008). Whether cell sorting is a conserved morphogenetic behavior during mammalian gastrulation remains to be tested.

Our knowledge of human gastrulation has largely been derived from studies in model organisms, including mouse and, more recently, cyno monkey (Ma et al., 2019; Nakamura et al., 2016; Niu et al., 2019). Ethical guidelines limit human embryo studies to 14 days post fertilization (dpf) or gastrulation onset (Pera, 2017), highlighting the need for alternative in vitro models. Demonstrating that embryonic stem cells (ESCs) can model aspects of in vivo gastrulation, mouse ESCs cultured in appropriate conditions, differentiate into the three germ layers and recapitulate aspects of spatial patterning, axis formation, symmetry breaking, self-organization, and polarization of gene expression (Beccari et al., 2018; Fuchs et al., 2012; Harrison et al., 2017; Kopper et al., 2010; Morgani et al., 2018; Sozen et al., 2018; ten Berge et al., 2008; Turner et al., 2017; van den Brink et al., 2014). Similarly, human ESCs (hESCs) may be used to model aspects of human gastrulation. Warmflash et al., 2014 reported that hESCs, cultured in confined micro-discs of extracellular matrix (ECM) and stimulated with BMP4, can reproducibly differentiate into radially organized cellular rings, expressing markers of ectoderm, mesoderm, endoderm, and TE, arranged from the center outwards, respectively. Mesendodermal cells in these cultures exhibit features of EMT, reminiscent of PS formation (Martyn et al., 2019a; Warmflash et al., 2014). However, the cellular complexity of these 2D micropatterned gastruloids remains to be determined.

Here we adapted the 2D micropatterned gastruloid culture (Warmflash et al., 2014) and validated that hESCs in micro-discs, upon BMP4 treatment, reproducibly form radially organized germ layers and ExE-like cells. Single-cell RNA sequencing (scRNA-seq) analyses revealed the formation in gastruloids of seven cell types, including epiblast, ectodermal, two mesodermal, and endodermal clusters, as well as previously undescribed cell types in the micropatterned gastruloids, PGC-like, and ExE-like that is transcriptionally similar to TE and amnion. Cross-species comparisons suggest that human gastruloids correspond to early-mid gastrula stage, based on the high resemblance in cellular composition and gene expression to E7.0 mouse and 16 dpf cyno monkey gastrulae. Finally, dissociated gastruloid cells displayed motility and cell sorting behaviors when re-seeded on ECM micro-discs: they tended to aggregate with similar, but segregate from unlike cell types in a mixed cell population. Thus, our work demonstrates that the in vitro hESC 2D micropatterned gastruloid system can generate cellular complexity of early-mid gastrulae with primate-specific transcriptomes and model conserved morphogenetic cell sorting behaviors. We also provide a rich resource for the transcriptomes of human embryonic and ExE cells relevant to the gastrulation stage.

## Results

### BMP4 differentiates hESCs in micro-discs into radial patterns of germ layers and ExE-like cells

**Figure 1.**
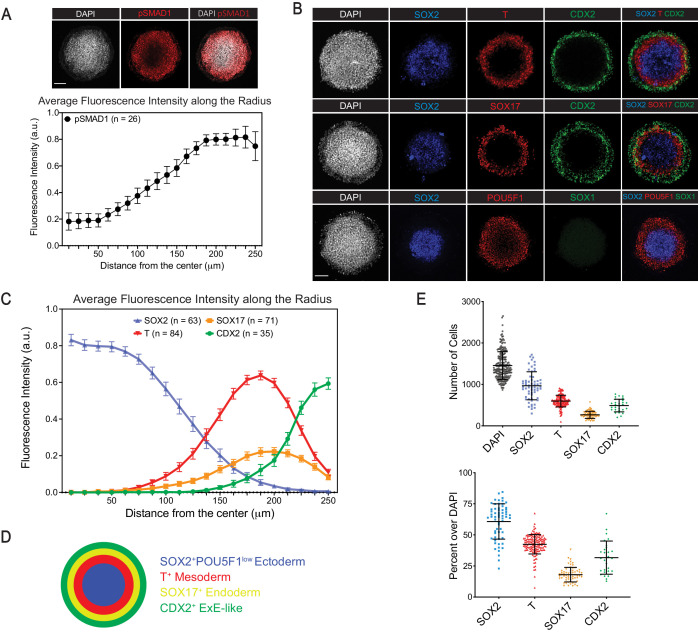
BMP4 induces differentiation of H1 hESCs in Matrigel micro-discs into radially-arranged germ layers and ExE-like cells after 44 hr. (**A**) Immunofluorescence images (top) and quantification of fluorescence intensity (bottom) for BMP4 downstream effector pSMAD1 relative to the DAPI fluorescence marking nuclei (N = 4 experiments; n = 26 gastruloids; pSAMD1 fluorescence intensity normalized against that of DAPI for each gastruloid along the radius and average normalized value of all gastruloids is shown; error bar represents standard error of the mean). (**B**) Immunofluorescence images of ectoderm marker SOX2, mesoderm marker T, endoderm marker SOX17, and TE marker CDX2. (**C**) Quantification of fluorescence intensity of indicated markers, normalized to DAPI and shown as averages along the radius of gastruloid (N = 2, 5, 6, 4 experiments; n = 63, 84, 71, 35 gastruloids, respectively for SOX2, T, SOX17, CDX2; fluorescence intensity of each marker is normalized against that of DAPI for each gastruloid along the radius and average normalized value of all gastruloids is shown; error bars represent standard error of the mean). (**D**) Schematic representation of cell differentiation in gastruloids. (**E**) Total number (top) and fraction (bottom) of cells expressing indicated markers in gastruloids (each dot represents a gastruloid; same number of gastruloids for each marker as in (**C**); error bars represent standard deviation). Scale bar is 100 µm.

To study aspects of human gastrulation, we adapted the hESC micropattern differentiation method developed by Warmflash et al., 2014. After treating H1 hESCs cultured on 500 µm-diameter ECM micro-discs with BMP4 for 44 hr (Figure 1—figure supplement 1), immunofluorescence analysis revealed a radial gradient of the downstream effector phosphorylated SMAD1, which declined from the edge to the center (Figure 1A). Consistent with previous work (Warmflash et al., 2014), we observed SOX2^+^POU5F1(OCT4)^-^ ectoderm, Brachyury or T^+^ mesoderm, SOX17^+^ endoderm, and CDX2^+^ ExE-like cells, arranged radially from center to edge (Figure 1B–D). Therefore, BMP4 treatment of H1 hESCs cultured on ECM micro-discs produced microcolonies termed ‘gastruloids’ with three prospective germ layers surrounded by a ring of ExE-like cells. We observed a similar radial differentiation pattern with H9 hESCs (Figure 1—figure supplement 2).

**Figure 1—figure supplement 1.**
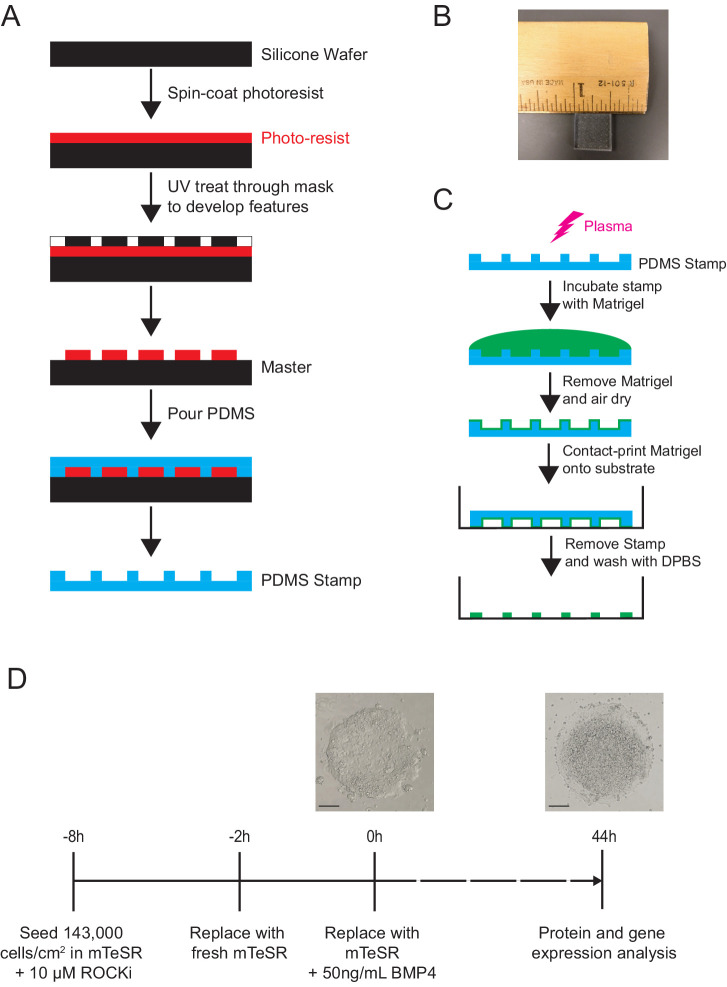
Protocol for microcontact printing method and micropatterned differentiation of gastruloids. (**A**) Schematic of protocol for fabricating PDMS (poly-dimethylsiloxane) stamp used in microcontact printing. (**B**) An image of a typical stamp used in microcontact printing with a ruler and one-inch mark for scale. (**C**) Schematic of protocol for Matrigel discs microcontact printing using PDMS stamp. (**D**) Protocol for BMP4-induced gastruloid differentiation.

**Figure 1—figure supplement 2.**
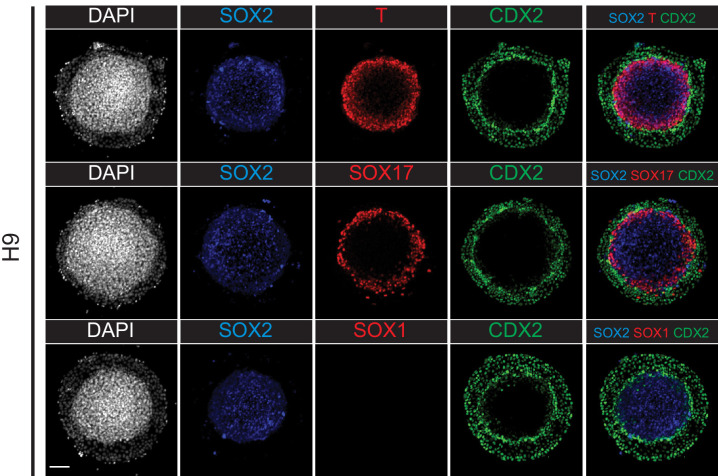
Immunofluorescence images of germ layer and TE markers in H9 hESC micropatterned gastruloid differentiation. Scale bar is 100 µm.

We assessed the number of cells expressing various markers by using a fully convolutional neural network analysis of immunofluorescence images (see ‘Materials and methods’) (He et al., 2019). The majority of H1 cells differentiated into SOX2^+^ ectodermal cells (61 ± 14%), followed by T^+^ mesodermal cells (42 ± 8%), CDX2^+^ ExE-like cells (32 ± 13%), and SOX17^+^ endodermal cells (18 ± 6%) (Figure 1E). Both the differentiation pattern and the proportion were consistent across multiple experiments (Figure 1C,E), highlighting the reproducibility of the micropattern culture system (Warmflash et al., 2014).

### Single-cell profiling reveals additional cell types relevant to gastrulation

**Figure 2.**
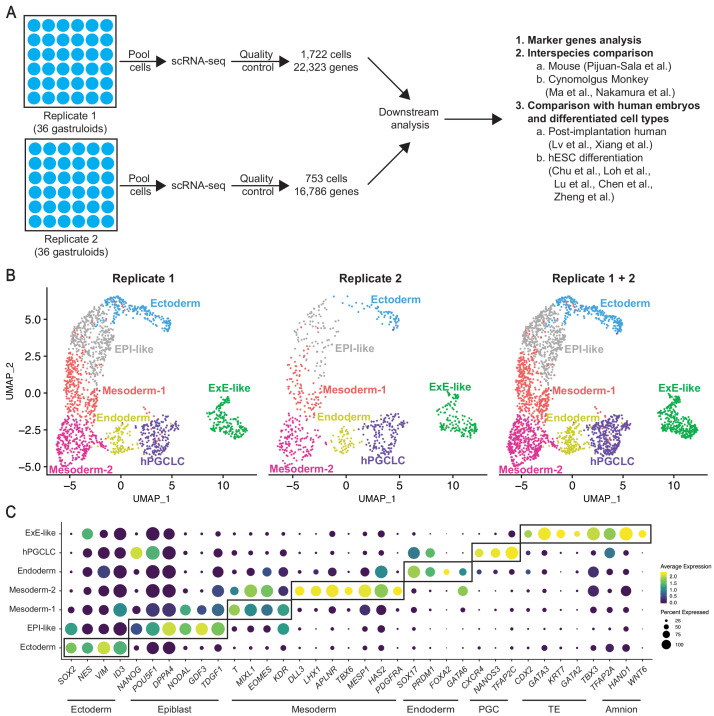
Single-cell RNA-seq profiling reveals cellular complexity of BMP4-induced gastruloids. (**A**) Schematic showing scRNA-seq workflow for gastruloids from two biological replicates. (**B**) UMAP display of seven cell clusters detected by unsupervised clustering in gastruloids replicate 1 (left), replicate 2 (middle), and both replicates combined (right). (**C**) Dot plot showing expression of canonical markers of ectoderm, epiblast, mesoderm, endoderm, PGC, TE, and amnion in the seven gastruloid clusters (PGC = Primordial Germ Cell; TE = Trophectoderm).

Our cell counting data indicated that the proportion of cells expressing ectoderm, mesoderm, endoderm, and ExE markers (61, 42, 18, and 32%, respectively) exceeded that of nuclei stained with DAPI or total cells per gastruloid (Figure 1E), suggesting that some cells co-expressed a combination of markers. Thus, we reasoned gastruloids contained more than four distinct cell types and transitional states, previously undescribed. To define all gastruloid cell types and determine their comprehensive transcriptomes, we performed scRNA-seq on two independent biological replicates; each replicate was pooled from 36 individual H1 hESC gastruloids. After quality control (see ‘Materials and methods’), we selected 1722 and 753 cells from replicates 1 and 2, respectively (Figure 2A, Figure 2—figure supplement 1). Using the Seurat R toolkit (Butler et al., 2018; Stuart et al., 2019), we integrated datasets from the two replicates using canonical correlation analysis and analyzed 2,475 cells expressing 23,271 genes. Unsupervised clustering revealed seven clusters (Figure 2B, Figure 2—figure supplement 2A). We found close correlation in gene expression between corresponding clusters of replicates 1 and 2 (Figure 2—figure supplement 2B). Likewise, all seven clusters overlapped well between the replicates (Figure 2B, Figure 2—figure supplement 2C). These data, along with immunofluorescence studies (Figure 1C,E), demonstrate that gastruloid differentiation is highly reproducible and consistent across independent biological replicates.

**Figure 2—figure supplement 1.**
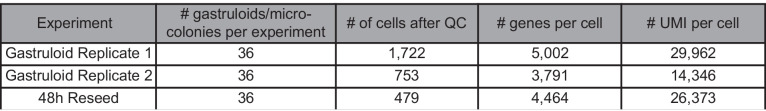
Selected parameters of scRNA-seq analysis.

**Figure 2—figure supplement 2.**
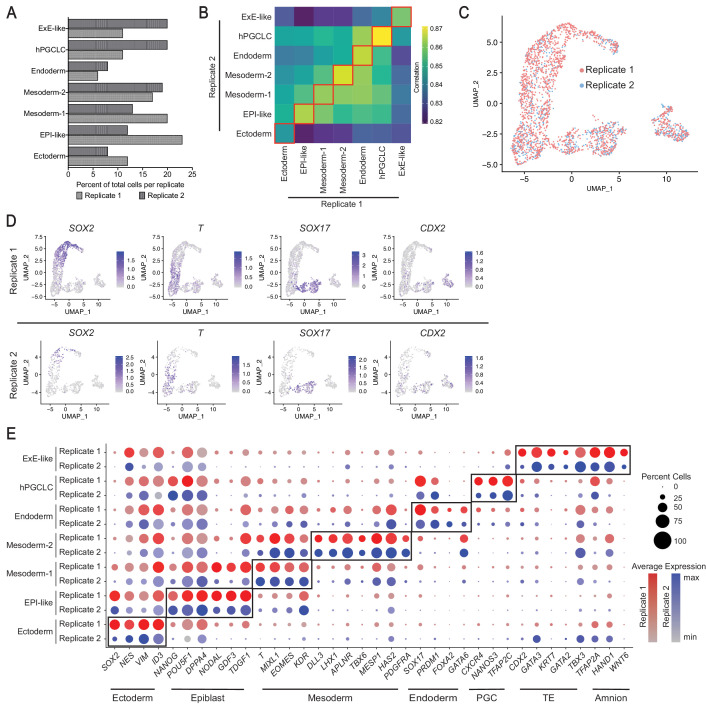
Comparison of gastruloid scRNA-seq datasets from two independent biological replicates. (**A**) Bar plot indicating relative proportion of cells in each cluster of replicates 1 and 2. (**B**) Heatmap showing correlation of average expression of 23,271 total genes between scRNA-seq clusters from replicates 1 and 2. (**C**) UMAP showing overlap of cells from replicates 1 and 2. (**D**) UMAP presenting expression of four markers used in immunofluorescence staining in replicates 1 and 2. (**E**) Dot plot showing expression of canonical markers of ectoderm, epiblast, mesoderm, endoderm, PGC, TE, and amnion in the seven gastruloid clusters in replicates 1 and 2 (PGC = Primordial Germ Cell; TE = Trophectoderm).

We used canonical markers, including those in the immunofluorescence study (Figure 2—figure supplement 2D), to annotate the seven clusters. We identified Ectoderm (*SOX2*^high^*POU5F1*^low^), Mesoderm-1 and -2 (*T^+^MIXL1^+^*), Endoderm (*SOX17^+^PRDM1^+^FOXA2^+^*), and ExE-like (*CDX2*^+^*GATA3*^+^), as expected from protein expression analysis (Figure 2B–C). Mesoderm-2 differs from Mesoderm-1 in the enrichment of markers for more mature mesoderm such as *MESP1*, *TBX6*, and *LHX1* (Figure 2C). Interestingly, we identified a cluster enriched in PGC markers, *NANOS3* and *TFAP2C* (Chen et al., 2019), suggesting that gastruloids may contain human PGC-like cells (hPGCLCs) (Figure 2C), a cell type previously not described in the micropatterned gastruloid culture system. Furthermore, we discovered that the ExE-like cluster is enriched not only in TE markers, *CDX2* and *GATA3*, as previously reported, but also in amnion markers such as *TFAP2A*, *WNT6*, and *HAND1* (Ma et al., 2019; Niu et al., 2019; Zheng et al., 2019). All markers used to annotate clusters were well-represented in both replicates (Figure 2—figure supplement 2E). Overall, these data showed reproducibility between the two scRNA-seq replicates and the identification of seven clusters based on canonical markers. We used the combined dataset of replicates 1 and 2 in downstream analyses unless otherwise noted.

We next wished to probe further the characteristics of the gastruloid clusters beyond canonical marker expression. First, what stage of the gastrulation in vivo does the gastruloid platform model? Second, can the gastruloids model conserved mechanisms of gastrulation and reveal its primate and/or human-specific features? Third, can the gastruloid platform be used to discover new mechanisms of human gastrulation? Thus, we queried gastruloid cellular identities in an unbiased and comprehensive manner using published datasets (Figure 2A), including gastrulating mouse embryos (Pijuan-Sala et al., 2019), post-implantation primate cyno monkey embryos (Ma et al., 2019; Nakamura et al., 2016), in vitro cultured human embryos (Lv et al., 2019; Xiang et al., 2020), and hESC-differentiated cell types (Chen et al., 2019; Chu et al., 2016; Loh et al., 2016; Lu et al., 2018; Zheng et al., 2019).

### Cross-species comparisons suggest similarity of gastruloid to early-mid gastrula stage

We performed a cross-species comparison with scRNA-seq data from gastrulating mouse embryos at E6.5, 6.75, 7.0, 7.25, and 7.5 (Pijuan-Sala et al., 2019) and in vitro cultured primate cyno monkey embryos at 11, 12, 13, 14, 16, and 17 dpf (Ma et al., 2019). Briefly, gastruloid and mouse or monkey data at all indicated stages were first integrated. For each gastruloid cell, we computed cell type or stage prediction scores based on the labels of its nearest neighbors in the reference mouse or monkey dataset (Stuart et al., 2019). The reference cell type or stage with the highest prediction score was assigned as the predicted cell type or stage for each gastruloid cell (see ‘Materials and methods’). Thus, each gastruloid cell contains labels for the original cluster annotation based on marker expression (Figure 2B–C) and predicted (or projected) mouse or monkey cell types and stages.

**Figure 3.**
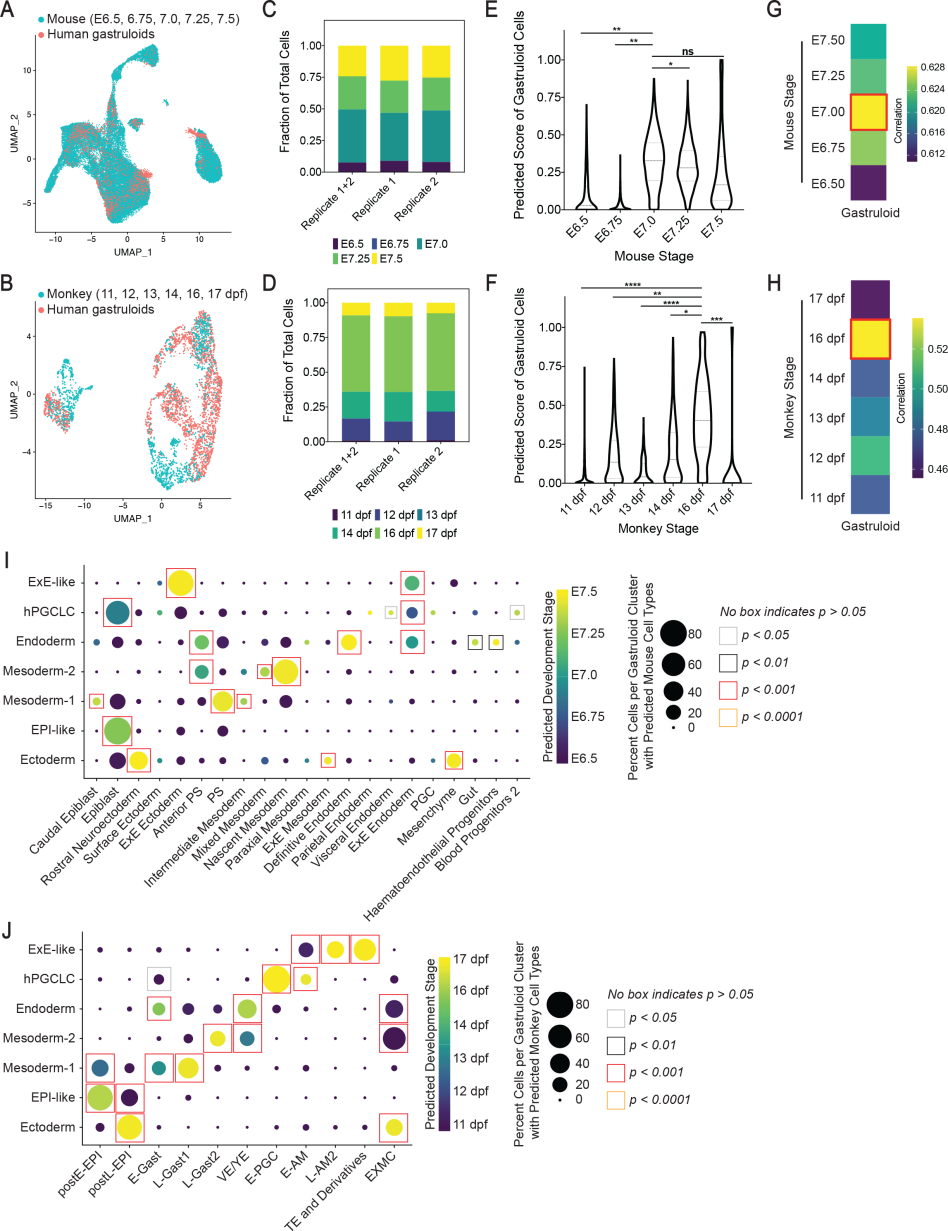
Cross-species comparison with mouse and cyno monkey gastrulating embryo. (**A, B**) UMAP displaying overlap of cells in gastruloids and mouse (**A**), and monkey (**B**) at indicated gastrulation stages. (**C, D**) Bar plots showing composition of predicted mouse (**C**) and monkey (**D**) cell stages in all gastruloids, replicate 1, and replicate 2. (**E, F**) Violin plots showing predicted score of mouse (**E**) and monkey (**F**) cell stages in gastruloid cells (ns = not significant; *, **, ***, **** indicates p<0.05, 0.01, 0.001, 0.0001, respectively, one-way ANOVA with Tukey’s multiple comparisons test; dash lines indicate 1 st, 2nd, and 3rd quartiles). (**G, H**) Gene expression correlation between gastruloids and mouse (**G**) and monkey (**H**) developmental stages using top 500 shared highly variable genes. (**I, J**) Dot plots showing predicted mouse (**I**) and monkey (**J**) cell types and stages in gastruloid clusters (Statistical significance of gastruloid cell types’ correspondences to monkey or mouse cell types is calculated by randomized permutation test; PS, primitive streak; postE/L-EPI, post-implantation early/late epiblast; E/L-Gast, early/late gastrulating cells; EXMC, ExE mesenchyme; VE/YE, visceral/yolk sac endoderm; E-PGC, early PGC; E/L-AM, early/late amnion; TE, trophectoderm).

We found that gastruloid data overlapped with specific mouse and monkey cells during gastrulation (Figure 3A–B). The composition of predicted cell types in gastruloids showed high similarity to E7.0 mouse and 16 dpf monkey in terms of cell type composition (Figure 3—figure supplement 1), implying that gastruloids represent early-mid gastrula stage in cellular composition. Alternatively, projection of mouse or monkey cell stages onto gastruloid data showed that the greatest number of gastruloid cells were predicted to be E7.0 mouse (42%; Figure 3C) and 16 dpf monkey (54%; Figure 3D). Similarly, predicted mouse or monkey cell stage scores for individual gastruloid cells revealed the highest score for E7.0 mouse and 16 dpf monkey (Figure 3E–F). Corroborating these findings, we found the closest correlation in gene expression between gastruloids and E7.0 mouse or 16 dpf monkey (Figure 3G–H). Emphasizing the reproducibility of the micropatterned gastruloid system, we found a strikingly similar composition of predicted mouse or monkey cell stages and types between the two gastruloid replicates (Figure 3I–J, Figure 3—figure supplement 2). Furthermore, 16 dpf monkey cells also scored the highest for E7.0 mouse (Figure 3—figure supplement 3A), and their gene expression correlated closely to E7.0 mouse (Figure 3—figure supplement 3B). These data suggest that the developmental stage of 16 dpf cyno monkey corresponds to that of E7.0 mouse, and that gastruloids resemble cell stages at both E7.0 mouse and 16 dpf cyno monkey gastrulae. Based on these results, we posit that micropatterned gastruloids after 44 hr of BMP4 differentiation resemble early-mid gastrula stage.

**Figure 3—figure supplement 1.**
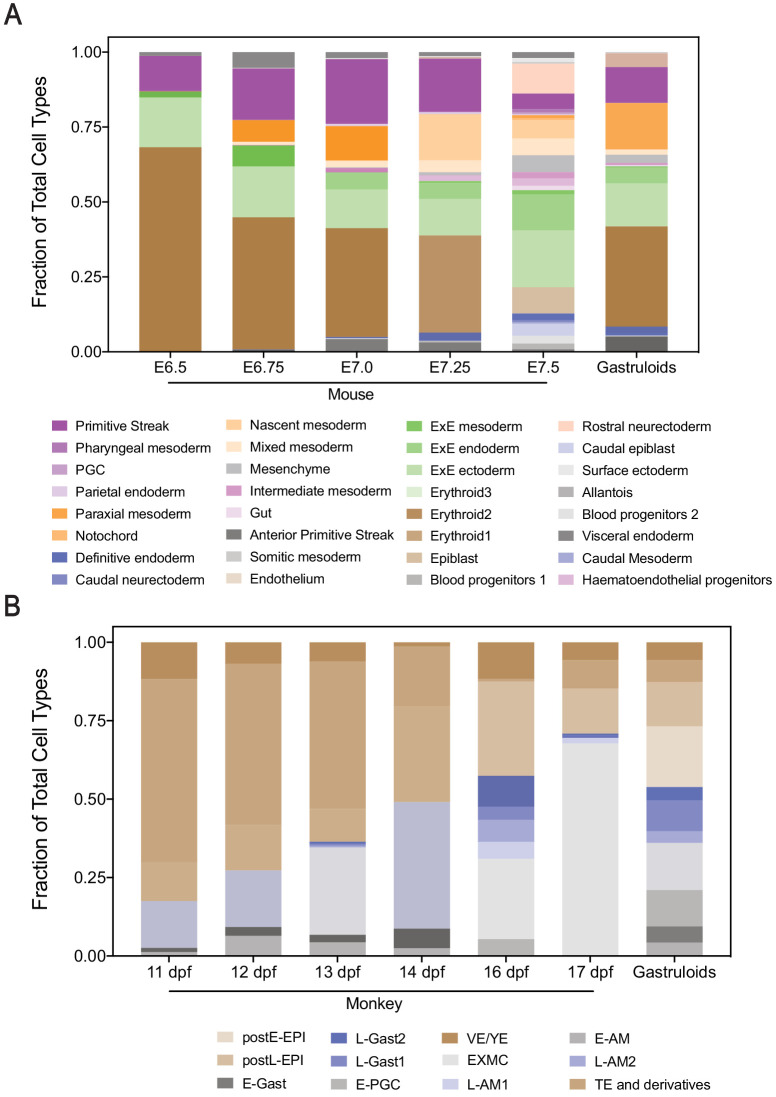
Gastruloids exhibit similar cell type composition profile to early-mid mouse and cynomolgus monkey gastrula stages. (**A, B**) Bar plots showing cellular composition of mouse (**A**) and cynomolgus monkey (**B**) embryos at indicated stages, and gastruloids with predicted cell types (each color bar represents a cell type).

**Figure 3—figure supplement 2.**
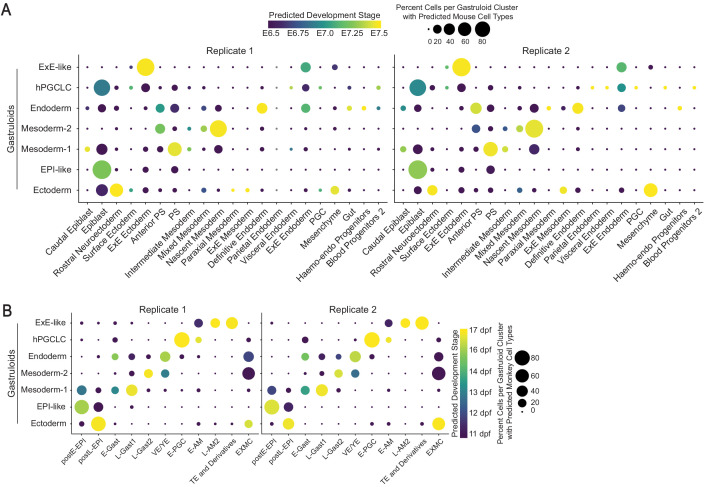
Species comparison shows high similarity between the two gastruloid replicates. (**A, B**) Dot plots showing predicted mouse (**A**) and cynomolgus monkey (**B**) cell types and stages in each cluster of gastruloid replicate 1 and replicate 2.

**Figure 3—figure supplement 3.**
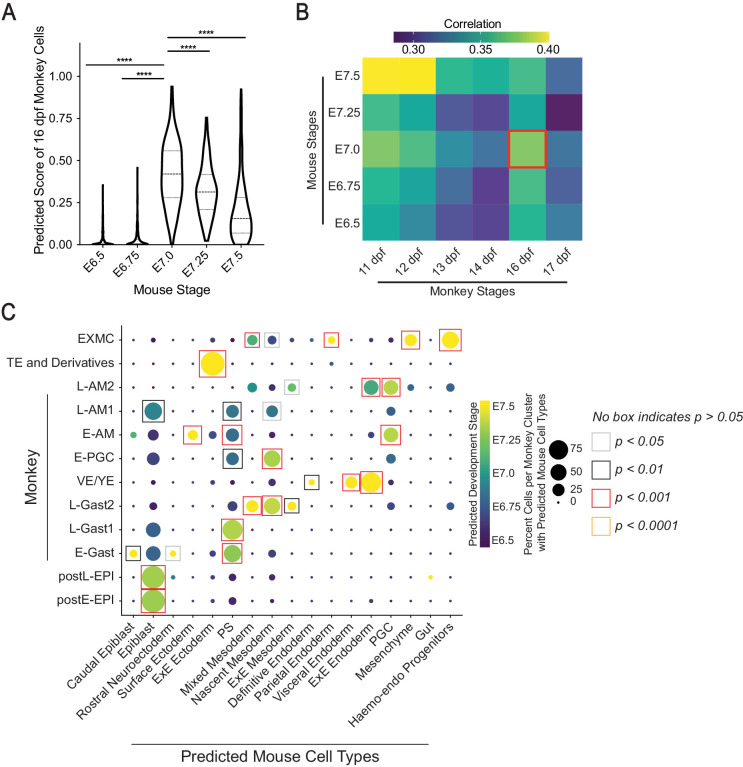
Species comparison between mouse and cynomolgus monkey gastrulating embryos. (**A**) Violin plot showing predicted score of mouse development stages in 16 dpf cynomolgus monkey cells (**** indicates p<0.0001, using one-way ANOVA with Tukey’s multiple comparisons test; dash lines indicate 1st, 2nd, and 3rd quartiles). (**B**) Correlation of average expression of top 500 shared highly variable genes between indicated mouse and cynomolgus monkey developmental stages. (**C**) Dot plot showing predicted mouse cell types and stages in cynomolgus monkey cell types (Statistical significance of monkey cell types’ correspondences to mouse cell types is calculated by randomized permutation test).

**Figure 3—figure supplement 4.**
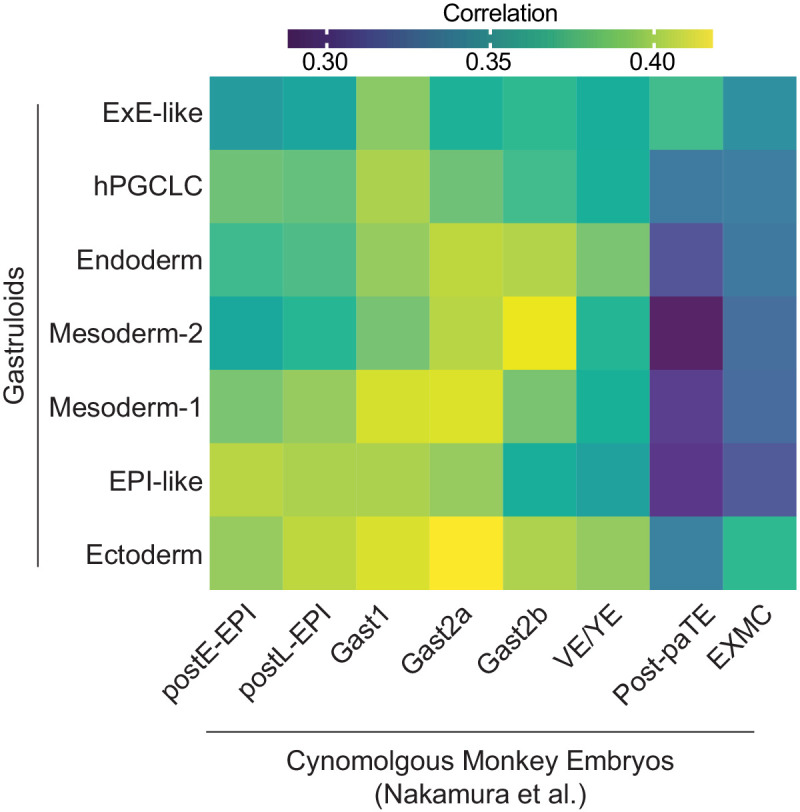
Heatmap of average gene expression correlation between gastruloids and cynomolgus monkey embryos from a separate study. (Based on 492 shared highly variable genes between gastruloids and cynomolgus monkey dataset from Nakamura et al., 2016).

### EPI-like cluster with characteristics of post-implantation epiblast

**Figure 4.**
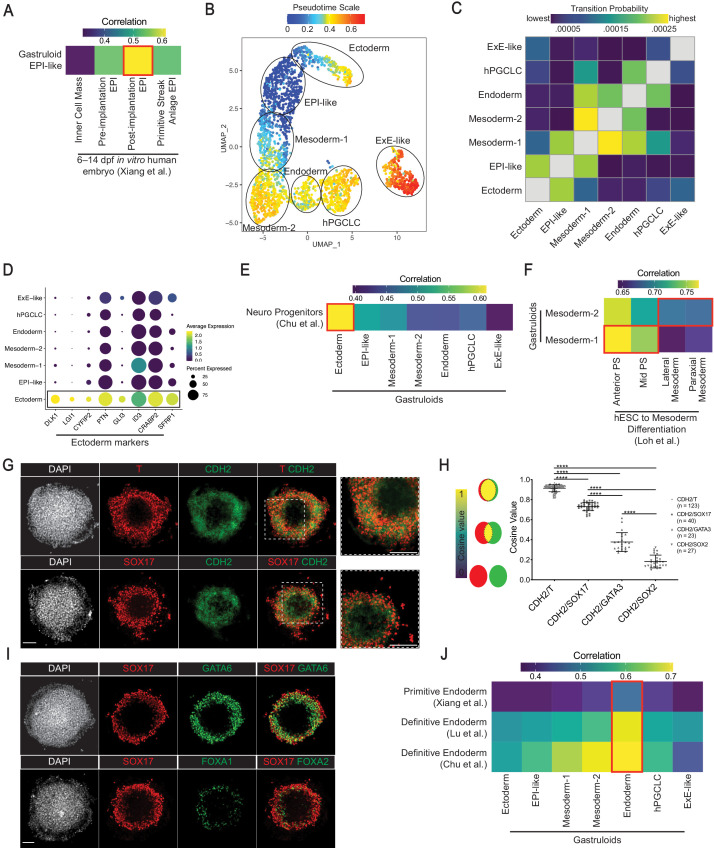
Characterization of gastruloid EPI-like, Ectoderm, Mesoderm, and Endoderm clusters. (**A**) Average expression correlation of 434 shared highly variable genes between the gastruloid EPI-like cluster and epiblast-related cell types from in vitro cultured human embryos. (**B**) UMAP displaying pseudotime overlay in gastruloid clusters. (**C**) Heatmap illustrating probabilities of a gastruloid cluster transitioning into other clusters. (**D**) Dot plot showing expression of indicated neural and nonneural ectoderm markers. (**E**) Average expression correlation of 546 shared highly variable genes between gastruloid clusters and hESC-differentiated neuro progenitors. (**F**) Average expression correlation of 323 shared highly variable genes between gastruloid Mesoderm-1 and -2 clusters, and hESC-differentiated PS- and mesoderm-related cell types. (**G**) Immunofluorescence images of CDH2 and indicated markers in gastruloids. (**H**) Cosine similarity analysis of expression domains between CDH2 and indicated markers (each dot represents a pair of indicated markers in a gastruloid; number of gastruloids used for each pair of indicated makers shown right of the chart; ****p*<*0.0001, one-way ANOVA with Tukey’s multiple comparisons test; error bars represent standard deviation). (**I**) Immunofluorescence images of primitive endoderm marker GATA6 and definitive endoderm maker FOXA2 in gastruloids. (**J**) Average gene expression correlation between gastruloid clusters and hESC-differentiated definitive endoderm, and primitive endoderm from in vitro cultured human embryos (based on 434, 374, and 546 shared highly variable genes with Xiang et al., Lu et al., and Chu et al., respectively). Scale bar is 100 µm.

Our scRNA-seq analyses defined a cell cluster enriched for human epiblast markers, *DPPA4*, *NANOG*, *POU5F1*, *SOX2*, *GDF3*, *NODAL*, and *TDGF1* (Petropoulos et al., 2016; Xiang et al., 2020; Figure 2C). Strong expression of *NODAL*, *GDF3*, and *TDGF1* suggested activity of NODAL signaling, which is known to posteriorize epiblast and is an evolutionarily conserved mesendoderm inducer from fish to mammals (Brennan et al., 2001; Schier, 2009). Projecting mouse (Pijuan-Sala et al., 2019) or monkey (Ma et al., 2019) cell type labels onto the gastruloid data showed that the majority of cells in the EPI-like cluster was predicted to correspond to mouse epiblast (86%; Figure 3I), and monkey post-implantation epiblast (postE-EPI, 71% and postL-EPI, 28%; Figure 3J). We also found that monkey postE-EPI and postL-EPI are predicted to correspond to mouse epiblast cells (Figure 3—figure supplement 3C). Moreover, the gastruloid EPI-like cluster exhibited the closest correlation in average gene expression to monkey postE-EPI and postL-EPI from another study (Nakamura et al., 2016; Figure 3—figure supplement 4), and in vitro cultured 6–14 dpf human embryo (Xiang et al., 2020; Figure 4A). These results suggest that the EPI-like cluster is transcriptionally similar to human, monkey, and mouse post-implantation epiblast.

**Figure 4—figure supplement 1.**
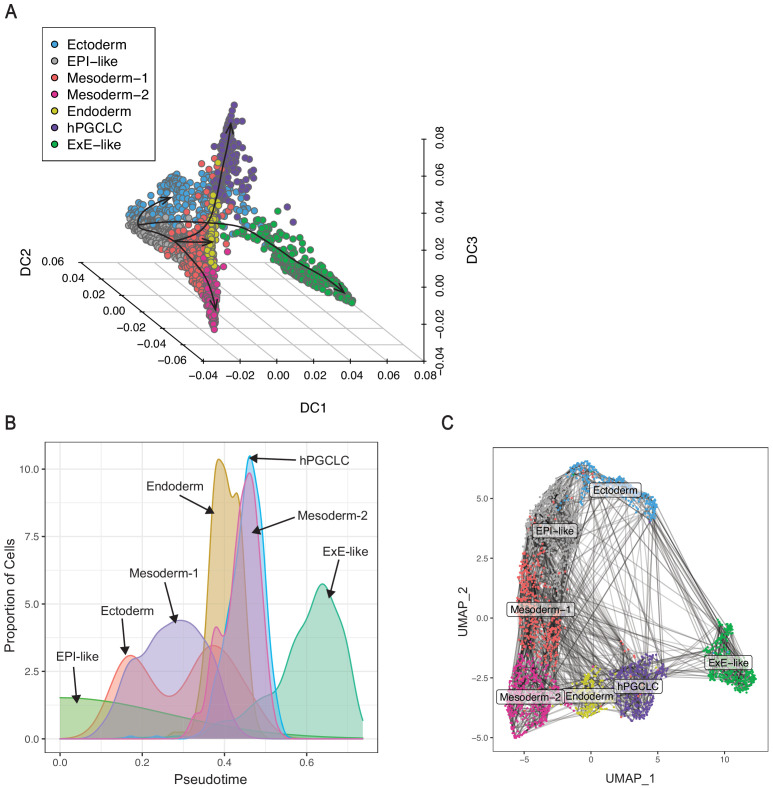
Pseudotime and transitional probability analyses in gastruloid clusters based on diffusion maps. (**A**) 3D plot showing gastruloid clusters in the first three diffusion coordinates (arrow lines indicate plausible differentiation trajectories). (**B**) Chart illustrating proportion of cells from all gastruloid clusters along the pseudotime. (**C**) UMAP plot showing all gastruloid clusters overlaid with transition probabilities (each line indicates transition probability between pairs of cells; 10,000 highest cell-to-cell transition probabilities shown).

**Figure 4—figure supplement 2.**
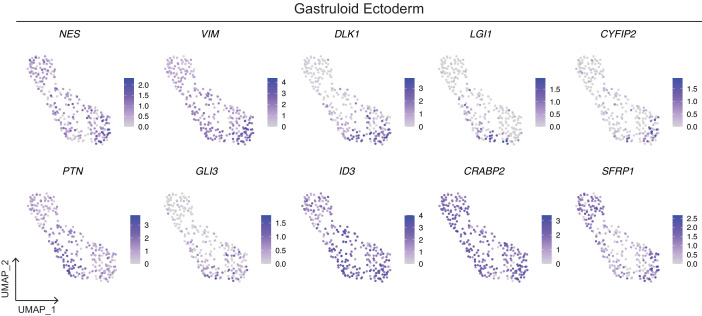
Expression of neural and surface ectoderm markers in the gastruloid Ectoderm cluster.

**Figure 4—figure supplement 3.**
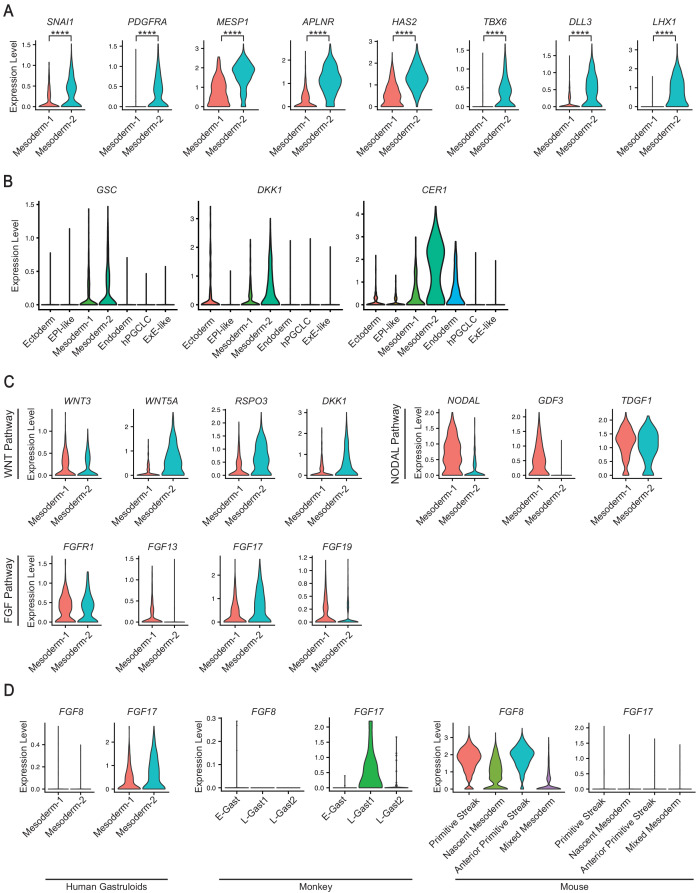
Expression of selected markers in gastruloid mesoderm clusters. (**A**) Violin plot showing expression of indicated mesoderm markers in the gastruloid Mesoderm-1 and -2 clusters (**** indicates p<0.0001 using Mann-Whitney test). (**B**) Violin plot showing expression of indicated markers for organizer cells in gastruloids. (**C**) Violin plots showing expression of WNT, NODAL, and FGF pathway components in the gastruloid Mesoderm-1 and -2 clusters. (**D**) Violin plots showing expression of *FGF8* and *FGF17* in the gastruloid Mesoderm-1 and -2 clusters, monkey gastrulating cells, and mouse primitive streak and mesoderm cells (E/L-Gast = early/late gastrulating).

**Figure 4—figure supplement 4.**
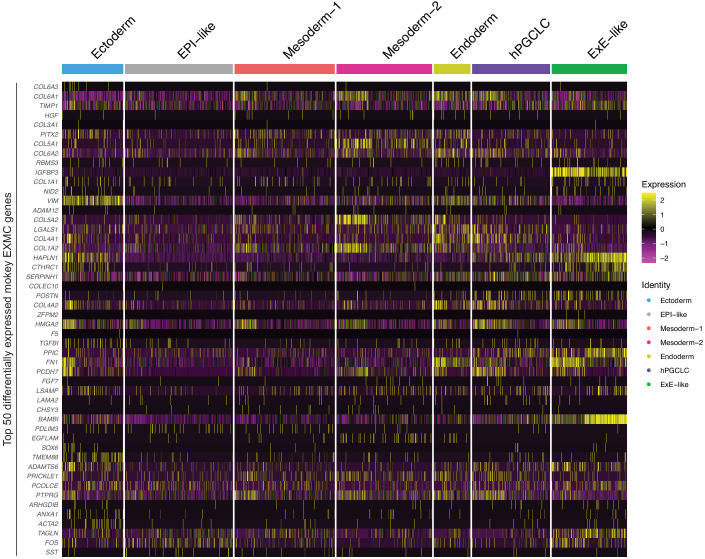
Expression of top 50 differentially expressed monkey EXMC markers in gastruloids.

**Figure 4—figure supplement 5.**
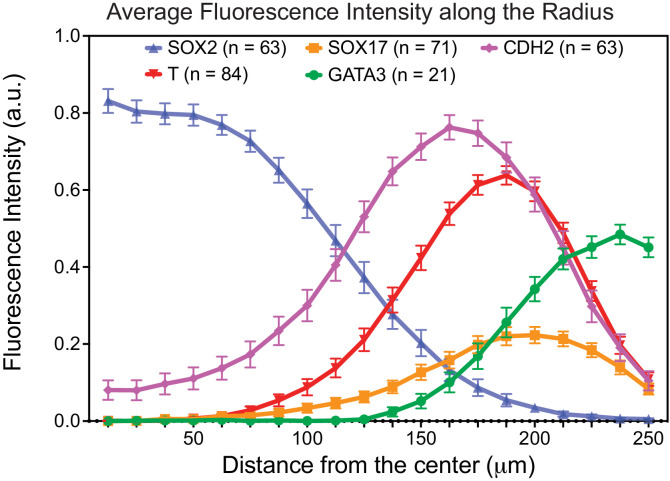
Overlap of CDH2 expression with T and SOX17 expression in gastruloids. (N = 2, 5, 6, 1, five experiments; n = 63, 84, 71, 21, 63 gastruloids, respectively for SOX2, T, SOX17, GATA3, CDH2; fluorescence intensity of each marker is normalized against that of DAPI for each gastruloid along the radius and average normalized value of all gastruloids is shown; error bars represent standard error of the mean).

**Figure 4—figure supplement 6.**
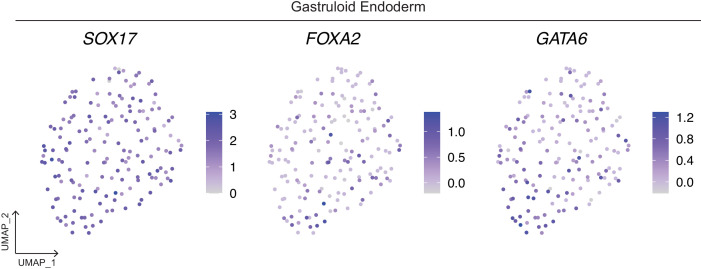
Expression of definitive endoderm marker *FOXA2* and primitive endoderm marker *GATA6* in the *SOX17^+^* gastruloid Endoderm cluster.

Using the R Bioconductor *destiny* package (Angerer et al., 2016), we calculated diffusion maps (Figure 4—figure supplement 1A) to investigate pseudotime and transition probabilities among gastruloid clusters. Pseudotime placed germ layer-related Ectoderm and Mesoderm-1 immediately after the EPI-like cluster (Figure 4B, Figure 4—figure supplement 1B), which also had the highest probability to transition to Ectoderm and Mesoderm-1 (Figure 4C, Figure 4—figure supplement 1C). Therefore, our results are consistent with cells in the EPI-like cluster representing precursors of those in Ectoderm and Mesoderm-1 clusters, similar to epiblast cells being precursors of the germ layers.

### Ectodermal cluster expressing nonneural and neural ectoderm markers

Cells in the *SOX2*^high^*POU5F1*^low^ Ectoderm cluster expressed neuroectoderm markers, *NES* (Lendahl et al., 1990), *VIM* (Schnitzer et al., 1981), *DLK1* (Surmacz et al., 2012), and *LGI1* (Tchieu et al., 2017). We also detected transcripts expressed in mouse ectodermal derivatives, *CYFIP2*, *PTN*, *GLI3*, *ID3*, *CRABP2*, and *SFRP1* (Pijuan-Sala et al., 2019; Figures 2C and 4D). While *CYFIP2, PTN, CRBP2*, and *GLI3* are expressed in rostral neuroectoderm, *CYFIP2* is also found in surface ectoderm of the E7.0 mouse embryo. *CRBP2* and *SFRP1* are spinal cord markers, while *PTN* and *GLI3* are forebrain/midbrain/hindbrain markers (Pijuan-Sala et al., 2019). The expression of these marker genes in the gastruloid Ectoderm, however, does not distinguish two distinct populations of surface and neural ectoderm, suggestive of prospective ectoderm identity in gastruloids (Figure 4—figure supplement 2). Cross-species comparison showed that cells in this cluster were predicted to be mouse rostral neuroectoderm (35%; Figure 3I) and monkey postL-EPI (66%; Figure 3J). Also suggesting that some cells have not fully differentiated into ectoderm, a portion of them had a predicted label for mouse epiblast (26%; Figure 3I). Interestingly, some cells were predicted as ExE mesenchyme in mouse (19%) and monkey (26%). Nonetheless, the average gene expression of this cluster showed the closest correlation to that of hESC-derived neuro progenitors (Chu et al., 2016; Figure 4E). Overall, these data suggest that this cluster resembles prospective ectoderm, expressing nonneural and neural ectoderm genes.

### Mesodermal clusters with characteristics of PS and nascent mesoderm

During gastrulation, mesoderm precursors within the PS undergo EMT, ingress through the PS, and then migrate to differentiate into paraxial, intermediate, and lateral plate mesoderm (Tam and Behringer, 1997). Our analyses revealed two presumptive mesoderm clusters, termed Mesoderm-1 and -2, both of which expressed markers of PS and mesoderm, *T*, *MIXL1*, and *EOMES* (Costello et al., 2011; Figure 2C). Interestingly, Mesoderm-1 expressed *T* at slightly higher levels than Mesoderm-2, suggesting that Mesoderm-1 may represent PS-like cells. Mesoderm-2 showed significantly higher transcript levels (p<0.001) of genes expressed in mesodermal cells that have already traversed through the PS, including *SNAI1,* and markers of lateral plate *PDGFRA*, *MESP1*, *APLNR*, and *HAS2* (Chan et al., 2013; Klewer et al., 2006; Vodyanik et al., 2010), paraxial *TBX6* and *DLL3* (Lam et al., 2014; Loh et al., 2016), and intermediate mesoderm *LHX1* (Lam et al., 2014; Figure 4—figure supplement 3A). Moreover, both clusters expressed transcripts encoding the organizer marker GSC, and associated secreted inhibitors DKK1 and CER1 (Martyn et al., 2018; Figure 4—figure supplement 3B). Pseudotime analysis placed Mesoderm-2 after Mesoderm-1, which in turn, was placed after EPI-like (Figure 4B, Figure 4—figure supplement 1B), suggesting that Mesoderm-1 represents cells in transition to Mesoderm-2. Consistent with this, the Mesoderm-1 cluster had the highest probability to transition to Mesoderm-2 (Figure 4C). Hence, the Mesoderm-2 cluster likely represents nascent mesoderm and/or precursors of differentiating mesodermal lineages.

Consistent with Mesoderm-1 and -2 resembling mouse PS and nascent mesoderm, respectively, cross-species comparison with mouse suggested that more Mesoderm-1 cells were predicted to correspond to PS (44% vs. 2% in Mesoderm-2) but more Mesoderm-2 cells to nascent mesoderm (71% vs. 14% in Mesoderm-1; Figure 3I). Similarly, Mesoderm-1 showed closer average gene expression correlation to hESC-differentiated PS cell types, but Mesoderm-2 to lateral and paraxial mesoderm (Loh et al., 2016; Figure 4F). Cross-species comparison with monkey indicated that Mesoderm-1 and -2 had predicted labels for early gastrulating (E-Gast, 17% vs. 0%) and late gastrulating cells (L-Gast1, 43% vs. 6%; L-Gast2, 2% vs. 21%; Figure 3J). We noted that a fraction of Mesoderm-2 was predicted as monkey ExE mesenchyme (EXMC,~50%), likely due to the transcriptional similarity of EXMC to gastrulating cells (Niu et al., 2019) and a relatively large fraction of EXMC cells in the original dataset (33% of total cells encompassing 12 cell types) (Ma et al., 2019). Furthermore, the top 50 differentially expressed markers of monkey EXMC were broadly expressed across gastruloid cell types (Figure 4—figure supplement 4), arguing against the formation of EXMC cell types in gastruloids. Corroborating our pseudotime analysis that Mesoderm-1 likely represents a transitional state from EPI-like cells, a fraction of Mesoderm-1 cells had predicted labels of mouse epiblast (23% vs. 0% in Mesoderm-2; Figure 3I), and monkey epiblast (postE-EPI and postL-EPI, 34% vs. 0% in Mesoderm-2; Figure 3J). Taken together, we interpret these data that gastruloid cultures give rise to mesodermal cells similar to monkey gastrulating cells as well as mouse PS and nascent mesoderm, and that gastruloid Mesoderm-1 may represent a transitional state to Mesoderm-2.

During gastrulation, nascent mesendodermal cells undergo EMT, which is marked by the upregulation of CDH2 (Radice et al., 1997). Substantiating previous reports of EMT signature in gastruloids (Martyn et al., 2019b; Warmflash et al., 2014), immunofluorescence experiments revealed distinct expression of CDH2 in T^+^ and SOX17^+^ mesendodermal cells (Figure 4G). Quantification of fluorescence intensity indicated that the expression domain of CDH2 primarily overlapped with that of T and SOX17 (Figure 4—figure supplement 5). As an independent approach, we utilized cosine similarity analysis to measure the spatial overlap in expression patterns between CDH2 and cell-type-specific markers (see ‘Materials and methods’). Cosine values range from 0 to 1, with 0 representing no overlap to one representing a complete overlap between expression patterns of two markers. As expected, we found the cosine value or overlap between expression patterns of CDH2 and T or SOX17 to be significantly higher than that between CDH2 and ectoderm marker SOX2 or ExE-like marker GATA3 (p<0.0001; Figure 4H). These results indicate that T^+^ mesodermal and SOX17^+^ endodermal cells upregulate CDH2, similar to ingressing mesodermal cells in fish, chick, and mouse gastrulae (Hatta and Takeichi, 1986; Radice et al., 1997; Warga and Kane, 2007).

Coincident with CDH2 upregulation in T^+^ mesodermal cells, our scRNA-seq analyses revealed expression of *FGFR1* (Figure 4—figure supplement 3C) and enrichment of its downstream target *SNAI1* (Warmflash et al., 2014) in Mesoderm-1 and −2 (Figure 4—figure supplement 3A). SNAI1 is a *CDH1* transcriptional repressor and evolutionarily conserved EMT inducer (Barrallo-Gimeno and Nieto, 2005), suggesting that EMT is mediated through a similar mechanism in gastruloids. We also found expression of components of FGF, WNT, and NODAL pathways (Figure 4—figure supplement 3C), suggesting activation of these pathways, which underlies the EMT induction in mouse (Nieto et al., 2016). We observed absent or low transcript levels of *FGF8* (Figure 4—figure supplement 3D), which is dispensable for EMT but required for cell migration away from the PS during mouse gastrulation (Sun et al., 1999). Instead, the gastruloid Mesoderm-1 and -2 clusters expressed *FGF17* (Figure 4—figure supplement 3D). Interestingly, *FGF8^low^FGF17^high^* signature was also found in the monkey but not in the mouse gastrulating cells (Figure 4—figure supplement 3D), suggesting that a different FGF ligand is responsible for promoting cell migration during human/primate gastrulation. Hence, we interpret these data that while gastruloid cells utilize evolutionarily conserved pathways to undergo EMT and cell migration, specific components involved may differ between human and mouse.

### Gastruloids contain cells resembling primitive and definitive endoderm

In gastruloids, we identified a *SOX17*^+^*PRDM1*^+^ presumptive Endoderm cluster, which expressed both primitive endoderm marker *GATA6* and definitive endoderm marker *FOXA2* (Figure 2C). However, *GATA6* and *FOXA2* expression was not mutually exclusive in the gastruloid Endoderm (Figure 4—figure supplement 6). Similarly, we detected immunofluorescence staining of GATA6 and FOXA2 in subsets of SOX17^+^ cells (Figure 4I). Projection of mouse cell type labels showed that the Endoderm cluster had predicted labels of definitive endoderm (23%), anterior PS (18%), ExE Endoderm (13%), and PS (12%; Figure 3I). Projection of cell type labels from the monkey dataset, which lacks definitive endoderm (Ma et al., 2019), showed that the Endoderm cluster had predicted labels of visceral endoderm (VE/YE, 36%), ExE mesenchyme (EXMC,31%), and gastrulating cells (E-Gast and L-Gast1/2, 28%; Figure 3J). Further comparison with 3D human embryo culture (Xiang et al., 2020) and hESC-derived definitive endoderm (Chu et al., 2016; Lu et al., 2018) showed that gastruloid Endoderm cluster exhibited close correlation in gene expression to both primitive and definitive endoderm (Figure 4J). Thus, we reasoned that the Endoderm cluster represents precursors or a mixture of both primitive and definitive endoderm.

### Gastruloids contain cells similar in gene expression profiles to primordial germ cells

**Figure 5.**
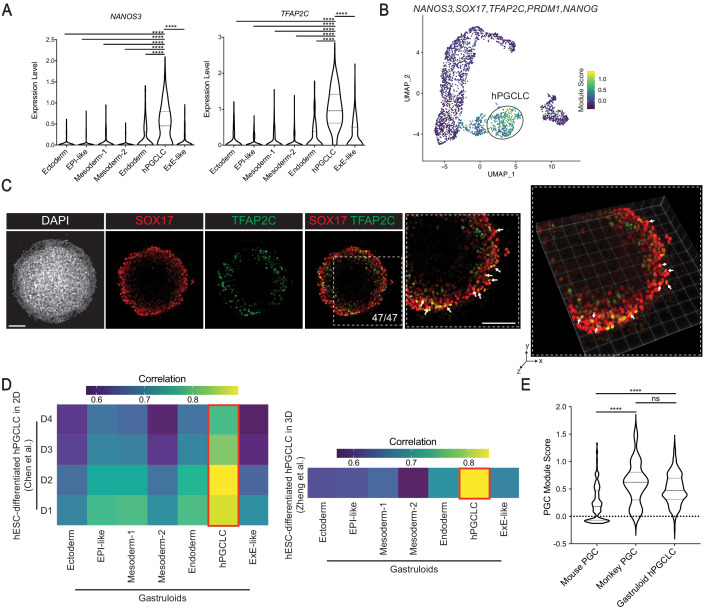
Characterization of gastruloid hPGCLCs. (**A**) Violin plot showing expression of PGC markers *NANOS3* and *TFAP2C* in gastruloid clusters (**** indicates p<0.0001; one-way ANOVA with Tukey’s multiple comparisons test; dash lines indicate 1st, 2nd, and 3rd quartiles). (**B**) UMAP showing module score of PGC signature *NANOS3, SOX17, TFAP2C, PRDM1,* and *NANOG* in gastruloid clusters. (**C**) Immunofluorescence images of indicated markers in 2D (left) and 3D (right) (arrows indicate selected TFAP2C^+^SOX17^+^ hPGCLCs). (**D**) Heatmap showing average gene expression correlation between gastruloid clusters and hESC-derived hPGCLC in 2D (left) and 3D (right) culture conditions (based on 1055 and 1129 shared highly variable genes with Chen et al. and Zheng et al., respectively). (**E**) Violin plot illustrating module scores of primate PGC signature *NANOS3, SOX17, TFAP2C, PRDM1,* and *NANOG* in mouse PGC, monkey PGC, and gastruloid hPGCLC (ns = not significant; ****p*<*0.0001, one-way ANOVA with Tukey’s multiple comparisons test; dash lines indicate 1st, 2nd, and 3rd quartiles). Scale bar is 100 µm.

The presence of hPGCLC has not been described in BMP4-differentiated gastruloids (Warmflash et al., 2014). Our scRNA-seq discovered a hPGCLC cluster significantly enriched for PGC markers *NANOS3* and *TFAP2C* (Chen et al., 2019; Sasaki et al., 2016) (p<*0*.0001; Figure 5A), suggesting that hPGCLCs arose in gastruloids. This cluster had gene expression profile, *SOX17^+^TFAP2C^+^PRDM1^+^POU5F1^+^NANOG^+^T^+^SOX2^low^* (Figure 5—figure supplement 1A), as reported for cyno monkey PGCs (Sasaki et al., 2016). The co-expression of *SOX17*, *TFAP2C*, *NANOG*, and *NANOS3* in hPGCLC was also reported in a recent study that implicated *TFAP2C* in *SOX17* regulation during human germline development (Chen et al., 2019). We calculated the PGC module score, the difference between the average expression of PGC markers (*NANOS3*, *SOX17*, *TFAP2C*, *PRDM1,* and *NANOG*) and that of randomly assigned genes (Butler et al., 2018; Tirosh et al., 2016), and found that the hPGCLC cluster scored the highest (Figure 5B). Validating scRNA-seq findings, we observed SOX17^+^TFAP2C^+^ cells in all gastruloids analyzed (47/47; Figure 5C, Figure 5—figure supplement 1B). We noted that TFAP2C is primarily co-expressed in SOX17^+^ cells, some in amnion marker TFAP2A^+^ cells but not in T^+^ mesodermal or GATA3^+^ ExE-like cells (Figure 5—figure supplement 1B–E).

**Figure 5—figure supplement 1.**
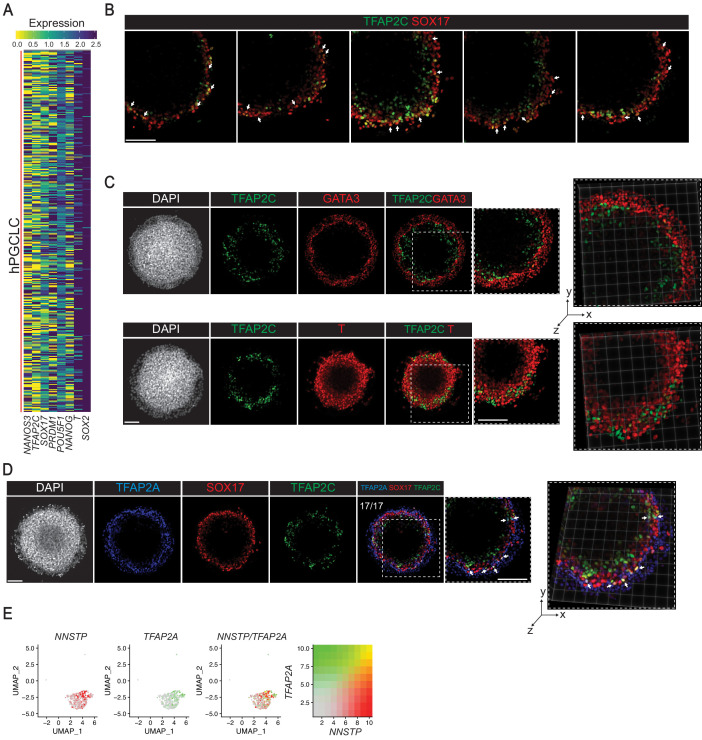
Expression of primate PGC markers in gastruloids. (**A**) Heatmap showing the expression of monkey early PGC signature in the gastruloid hPGCLC cluster. (**B**) Additional replicates of immunofluorescence images showing indicated markers (arrows indicate selected TFAP2C^+^SOX17^+^ hPGCLCs). (**C**) Immunofluorescence images of PGC marker TFAP2C, mesoderm marker T, and ExE marker GATA3 (note very few or no cell co-expresses TFAP2C and T or GATA3). (**D**) Immunofluorescence images of amnion marker TFAP2A in SOX17^+^TFAP2C^+^ hPGCLC (arrows indicate selected TFAP2A^+^SOX17^+^TFAP2C^+^ cells). (**E**) UMAP showing co-expression of amnion marker *TFAP2A* in *NNSTP* (*NANOS3^+^NANOG^+^SOX17^+^TFAP2C^+^PRDM1^+^*) cells in the scRNA-seq of the hPGCLC cluster.

Cross-species comparison indicated that gastruloid hPGCLCs have predicted labels of monkey early PGCs (80%; Figure 3J). Gastruloid hPGCLCs also showed the closest gene expression correlation to hESC-derived hPGCLCs in 2D (Chen et al., 2019) and 3D (Zheng et al., 2019) culture conditions (Figure 5D). Interestingly however, cross-species comparison with mouse showed that the hPGCLC cluster had predicted labels of epiblast (64%), ExE ectoderm (13%), and ExE endoderm (10%; Figure 3I). We reasoned the mismatch occurred because hPGCLC formation and gene expression diverge from mouse PGCs (Tan and Tee, 2019). Accordingly, we found that the PGC module score, based on primate PGC predictors, of monkey PGCs was similar to that of gastruloid hPGCLCs but significantly higher than that of mouse PGCs (p<0.0001; Figure 5E). Taken together, these findings suggest that gastruloids contain hPGCLCs that are transcriptionally similar to monkey and human PGCs.

### ExE-like cluster contains cells with gene expression profiles similar to TE and amnion

**Figure 6.**
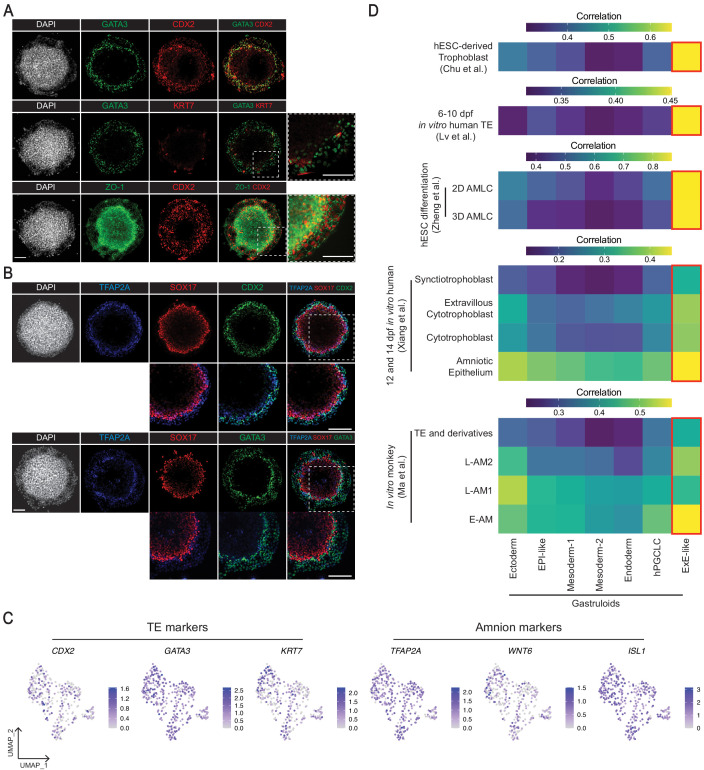
Characterization of the gastruloid ExE-like cluster. (**A, B**) Immunofluorescence images of TE makers GATA3 and CDX2 (**A**), and amnion marker TFAP2A (**B**) in gastruloids. (**C**) UMAP showing expression of TE markers *GATA3*, *CDX2*, *KRT7*, and amnion markers *TFAP2A*, *WNT6*, *ISL1* in the scRNA-seq of the ExE-like cluster. (**D**) Average gene expression correlation between gastruloid clusters and amnion or TE-related cell types from hESC differentiation, and in vitro cultured human and monkey embryos (based on 546, 524, 1,129, 434, and 371 shared highly variable genes with Chu et al., Lv et al., Zheng et al., Xiang et al., and Ma et al., respectively; AMLC = amnion-like cells; E/L-AM = early/late amnion). Scale bar is 100 µm.

We identified an ExE-like cluster enriched in TE markers *CDX2, GATA3,* and *KRT7* (Blakeley et al., 2015; Deglincerti et al., 2016). This cluster also expressed *GATA2* and *TBX3* (Figure 2C), genes required for trophoblast differentiation in mouse (Bai et al., 2013) and human (Lv et al., 2019), respectively. Validating scRNA-seq results, immunofluorescence staining showed CDX2, GATA3, and KRT7 expression in the outermost ExE-like cells. ZO-1 localization at the apical cell membranes indicated the epithelial character of these cells (Figure 6A). Furthermore, the ExE-like cluster showed the closest gene expression correlation to hESC-derived trophoblast (Chu et al., 2016) and human TE (Lv et al., 2019; Figure 6D). However, only a subset of ExE-like cells co-expressed KRT7 (Figure 6A), a marker for pan TE lineage in human (Deglincerti et al., 2016), suggesting the presence of TE and/or other ExE cellular subtypes.

Notably, the ExE-like cluster also expressed amnion markers *TFAP2A*, *HAND1*, and *WNT6* (Ma et al., 2019; Zheng et al., 2019; Figure 2C). Investigation at single-cell resolution revealed that ExE-like cells co-expressed TE markers GATA3 or CDX2 and amnion marker TFAP2A (Figure 6B–C), and cannot be distinguished into distinct TE or amnion cell types. Accordingly, comparison with published datasets suggested that the ExE-like cluster resembled both TE and amnion cells. Specifically, cross-species comparison with the mouse embryo dataset (Pijuan-Sala et al., 2019), which does not contain amnion cells, showed cells in the ExE-like cluster had predicted labels for ExE ectoderm (76%; Figure 3I), a derivative of mouse polar TE (Shahbazi and Zernicka-Goetz, 2018). Mouse ExE ectoderm was also predicted to resemble monkey TE in our cross-species analysis (Figure 3—figure supplement 3C). In contrast, comparison with the in vitro human amnion model (Zheng et al., 2019), lacking TE cells, showed the closest correlation between gastruloid ExE-like cells and hESC-derived amnion-like cells (AMLC; Figure 6D). Reconciling these results, comparison with monkey embryos, which possess both TE and amnion, showed gastruloid ExE-like cells had predicted labels for both TE (51%) and amnion (E-AM and L-AM2, 47%; Figure 3J). Likewise, the ExE-like cluster exhibited close gene expression correlation to monkey TE and amnion cells (Ma et al., 2019; Nakamura et al., 2016), and amniotic epithelium and trophoblast cell types from 12 and 14 dpf in vitro human embryos (Xiang et al., 2020; Figure 6D). Taken together, the ExE-like cluster likely contains both TE- and amnion-like cells, or an ExE-like cell type with TE and amnion gene expression signatures.

### BMP4-differentiated germ layer and ExE-like cells exhibit cell sorting behaviors

During amphibian and fish gastrulation, upon leaving the blastopore (PS equivalent), cells migrate to form distinct layers and organ rudiments. Cell sorting is thought to be a key driver of these early morphogenetic cell behaviors (Fagotto, 2015; Krens and Heisenberg, 2011; Winklbauer and Parent, 2017). Classic experiments of Holtfreter and colleagues revealed cell sorting behaviors, where dissociated cells from amphibian gastrulae were able to aggregate into the three germ layers in vitro (Townes and Holtfreter, 1955). Whether gastrulating mammalian cells exhibit such sorting behaviors remains to be tested.

**Figure 7.**
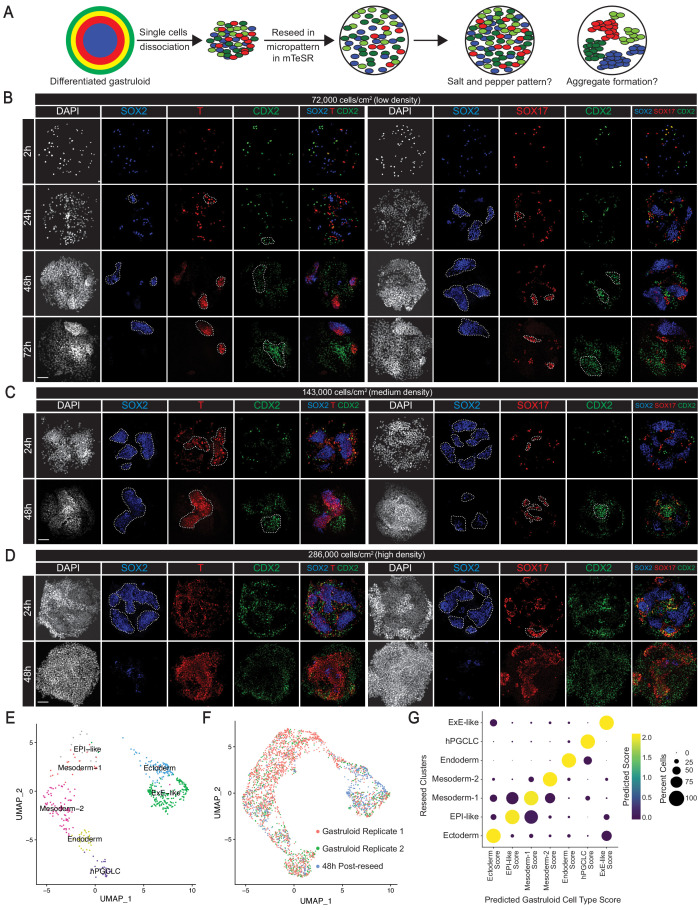
BMP4-differentiated germ layer and ExE-like cells exhibit cell sorting behaviors. (**A**) Schema of protocol for assaying cell sorting behaviors from dissociated gastruloids. (**B, C, D**) Immunofluorescence images of indicated markers in reseeded gastruloid cells at indicated timepoints and indicated cell densities (dashed lines indicate cellular aggregates). (**E**) UMAP showing the seven original gastruloid clusters in 48-hr post-reseed cells. (**F**) UMAP displaying overlap of gastruloid and reseeded cells. (**G**) Dot plot showing predicted cell type scores in reseeded clusters using the seven gastruloid clusters as reference. Scale bar is 100 µm.

We set out to investigate whether human gastruloid cells undergo cell sorting in vitro. H1 hESC gastruloids after 44 hr BMP4 treatment were dissociated into single cells. The resulting single-cell suspension was reseeded onto 500 µm-diameter ECM micro-discs at 72,000 cells/cm^2^ in mTeSR alone (Figure 7A). Time-lapse analyses of re-seeded cultures revealed that many cells were actively migrating on the ECM substrate and started to form aggregates by 15 hr (Figure 7—figure supplement 1A, Video 1). Immunostaining at 2 hr after reseeding revealed a random salt-and-pepper distribution of SOX2^+^, T^+^, SOX17^+^, and CDX2^+^ cells (Figure 7B). By 24 hr, SOX2^+^, T^+^, SOX17^+^, and CDX2^+^ cells tended to aggregate with cells expressing the same marker, suggesting the affinity to form homogeneous aggregates with same cell types. This tendency was more pronounced by 48 hr. By 72 hr, while aggregation of SOX2^+^, T^+^, and SOX17^+^ cells was still observable, CDX2^+^ cells mostly dominated individual microcolonies (Figure 7B, Figure 7—figure supplement 1B–C). At higher reseeded cell densities (143,000 cells/cm^2^ and 286,000 cells/cm^2^), we observed aggregation behavior earlier, within 24-hr post-reseeding. However, cells reseeded at the highest density overpopulated the culture discs and aggregation patterns were not as evident (Figure 7B–D). Moreover, immunofluorescence revealed a rapid loss of SOX2^+^ cells by 48 hr at the highest reseeded density, possibly due to overcrowding of other cell types (Amoyel and Bach, 2014; Figure 7—figure supplement 1C).

**Figure 7—figure supplement 1.**
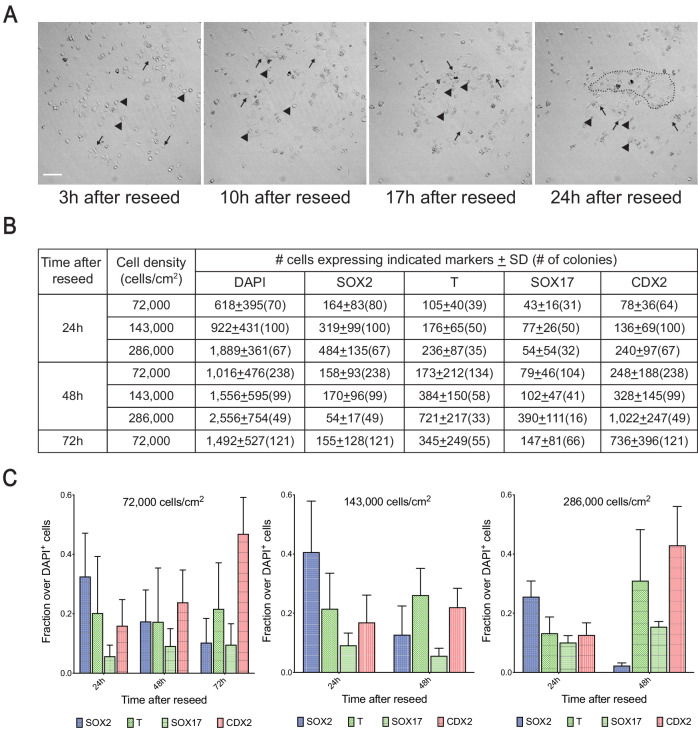
Visualization and quantification of reseeded cells. (**A**) Still time-lapse images of dissociated gastruloid cells reseeded in micropattern after 3, 10, 17, and 24 hr (arrow indicates motile cells, arrowhead indicates non-motile cells, dotted line indicates cellular aggregates). (**B, C**) Average number (**B**) and fraction (**C**) of dissociated and reseeded gastruloid cells positive for indicated markers at indicated timepoints and cell densities (number of reseeded colonies analyzed for each cell density and time point for (**B**) and (**C**) shown in parenthesis under each marker in (**B**); SD indicates standard deviation in (**B**); fraction in (**C**) calculated from cell numbers in (**B**); error bars indicate SD).

**Figure 7—figure supplement 2.**
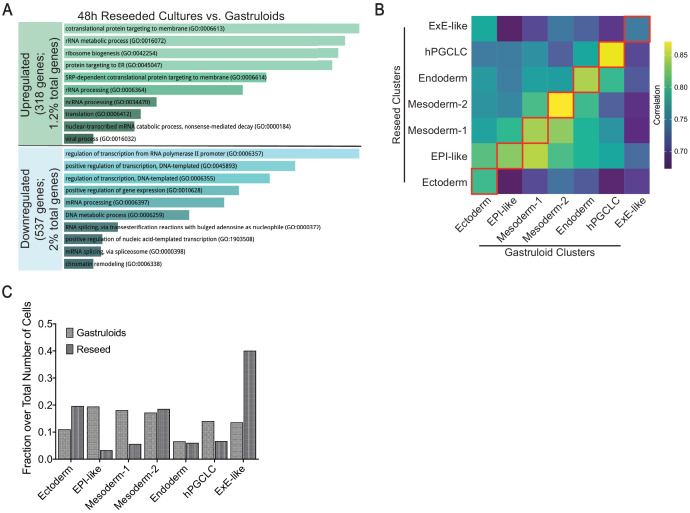
Reseeded cells retain transcriptional similarity to gastruloid cells. (**A**) Gene Ontology analysis of downregulated and upregulated genes between reseeded cultures and gastruloids. (**B**) Heatmap illustrating gene expression correlation of 2000 shared highly variable genes between clusters of reseeded and gastruloid cells. (**C**) Cellular composition of each cluster in gastruloids and reseeded cell population.

**Video 1.** Dissociated and reseeded gastruloid cells show motility. Time-lapse confocal series from 3 to 27 hr after reseeding of dissociated gastruloid cells in 500 µm ECM microdiscs.

We further characterized cell sorting behaviors in dissociated gastruloids at medium reseeding density (143,000 cells/cm^2^) since we observed aggregation behavior within 24 hr without overcrowding. ScRNA-seq analysis of gastruloid cells reseeded at medium density for 48 hr (479 cells expressing 16,253 genes; Figure 2—figure supplement 1) revealed the original seven gastruloid clusters (Figure 7E). The reseeded cells also overlapped closely with gastruloid cells (Figure 7F), arguing against significant changes in cell identities after reseeding. We noted 318 upregulated and 537 downregulated genes (1.2 and 2%, respectively, of total number of genes) in reseeded cells, but Gene Ontology analysis revealed that these genes are not associated with development or differentiation (Figure 7—figure supplement 2A). Further suggesting that gene expression did not alter significantly, reseeded clusters showed high gene expression correlation (Figure 7—figure supplement 2B), and predicted cell type scores (Figure 7G) to corresponding gastruloid clusters. Thus, these data argue against significant changes in cellular identity or gene expression between the reseeded and the original gastruloid cultures. However, we noted changes in proportions of the seven cell types (Figure 7—figure supplement 2C). Taken together, dissociated gastruloids, upon reseeding, maintained the seven original cell types for 48 hr, and showed cellular aggregation behavior, a characteristic of cell sorting capability.

### Selective cell sorting of reseeded gastruloid cells

**Figure 8.**
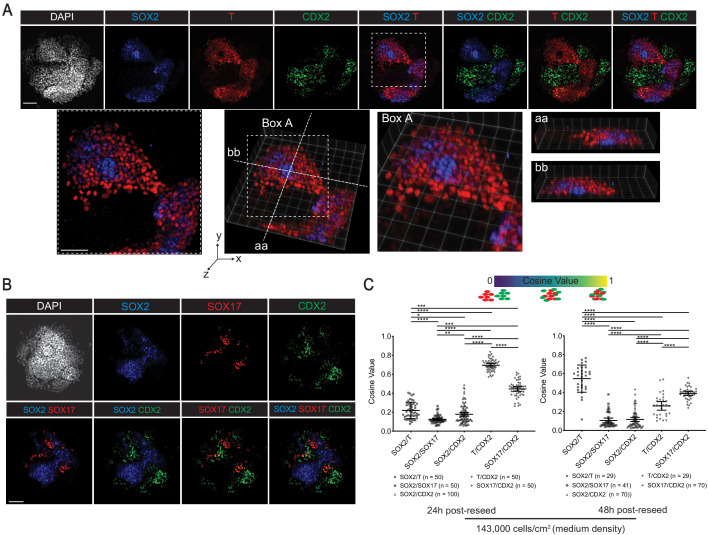
Reseeded gastruloid cells exhibit selective cell sorting behaviors. (**A, B**) Immunofluorescence images of indicated markers in 48 hr post-reseed cultures. (**C**) Cosine similarity analysis of expression domains between pairs of indicated markers (*, **, ***, **** indicates p<0.05, 0.01, 0.001, 0.0001, respectively, one-way ANOVA with Tukey’s multiple comparisons test; error bars represent standard deviation; each dot represents a pair of indicated markers in a reseeded colony; number of reseeded colonies used for each indicated pair of markers shown below the chart). Scale bar is 100 µm.

In a typical gastrula, including human, germ layers are arranged so that mesoderm is sandwiched between ectoderm and endoderm. Thus, ectoderm lies adjacent to mesoderm but is separated from endoderm (Gilbert, 2010). Similarly, in BMP4-derived gastruloids, the mesoderm ring separates ectoderm from endoderm (Warmflash et al., 2014; Figure 1B and D). We used SOX2, T, SOX17, and CDX2 to identify ectoderm, mesoderm, endoderm, and ExE-like cells, respectively, in our reseeding studies. However, we noted that SOX17 expression was shared by Endoderm and hPGCLC (Figures 4I and 5C). Immunofluorescence images of 48 hr reseeded cultures showed that SOX2^+^ cells tended to mix with T^+^ cells, with some cells overlapping on top of each other (Figure 8A, Figure 8—figure supplement 1A–B), but segregated from SOX17^+^ cells (Figure 8B and Figure 8—figure supplement 1A). Cosine similarity analysis, quantifying the spatial overlap between pairs of cell types, showed significantly higher cosine value between expression domains of SOX2 and T compared to that between SOX2 and SOX17 at all cell densities by 48 hr (p<0.0001; Figure 8C, Figure 8—figure supplement 2), suggesting that ectodermal cells readily associate with mesodermal cells but segregate from endodermal cells.

**Figure 8—figure supplement 1.**
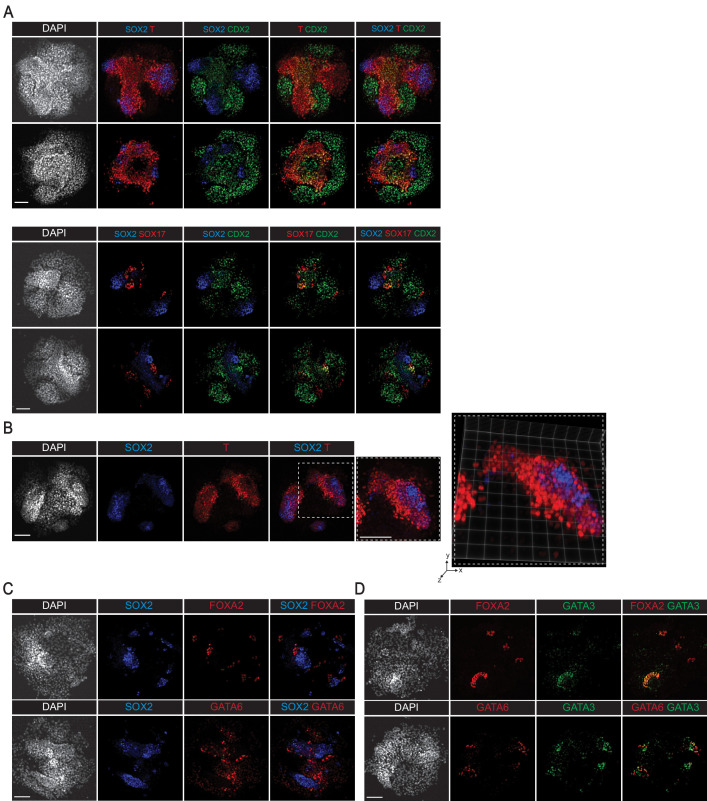
Immunofluorescence analyses of cell sorting behavior in reseeded cells. (**A**) Additional replicates of immunofluorescence staining showing indicated markers of dissociated and reseeded gastruloid cells after 48 hr (note mixing between SOX2^+^ and T^+^ cells, but segregation between SOX2^+^ and SOX17^+^ cells). (**B**) 2D and 3D-perspective images showing mixing between SOX2^+^ and T^+^ cells in dissociated and reseeded gastruloid cells after 48 hr. (**C, D**) Immunofluorescence images showing relative sorting between SOX2^+^ ectoderm cells (**C**) or GATA3^+^ ExE-like cells (**D**) and FOXA2^+^ definitive endoderm or GATA6^+^ primitive endoderm cells. Scale bar is 100 µm.

**Figure 8—figure supplement 2.**
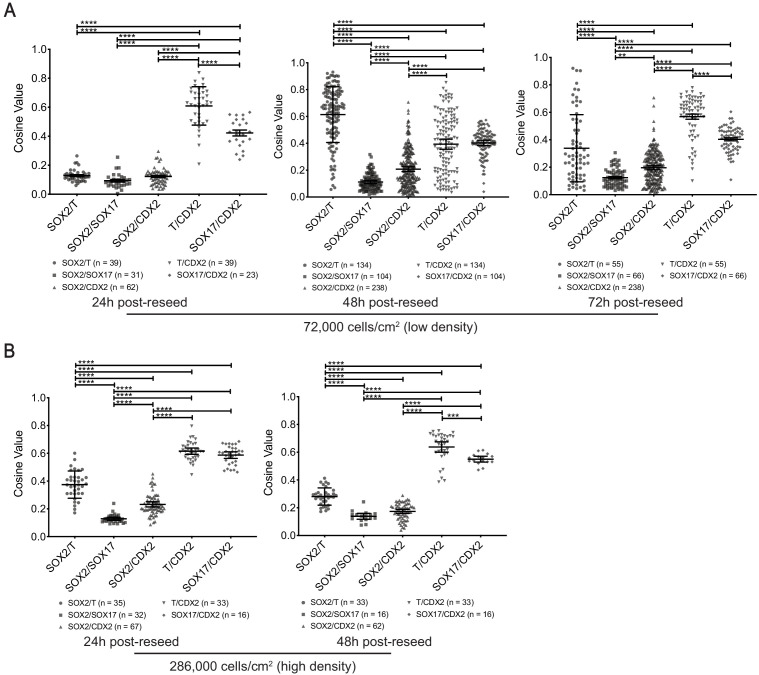
Cosine similarity or expression pattern overlap analysis between cell type markers in reseeded cells. (**A, B**) Cosine value depicting overlap between expression domains of pairs of indicated markers at indicated timepoints and cell densities (*, **, ***, **** indicates p<0.05, 0.01, 0.001, 0.0001, respectively, using one-way ANOVA with Tukey’s multiple comparisons test; error bars represent standard deviation; each dot represents a pair of indicated markers in a reseeded colony; number of reseeded colonies used for each indicated pair of markers shown below the chart).

**Figure 8—figure supplement 3.**
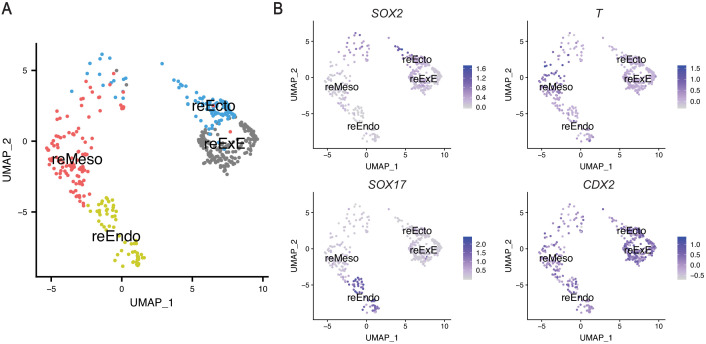
ScRNA-seq analysis of protein markers used in immunofluorescence studies of reseeded cells. (**A**) UMAP display of reseeded culture from dissociated gastruloids showing combined clusters with strong expression of *SOX2* (EPI-like and Ectoderm clusters into reEcto), *T* (Mesoderm-1 and -2 clusters into reMeso), *SOX17* (Endoderm and hPGCLC clusters into reEndo), and *CDX2* (ExE-like cluster into reExE). (**B**) UMAP depicting expression of genes encoding the protein markers used in immunofluorescence studies in reseed culture.

**Figure 8—figure supplement 4.**
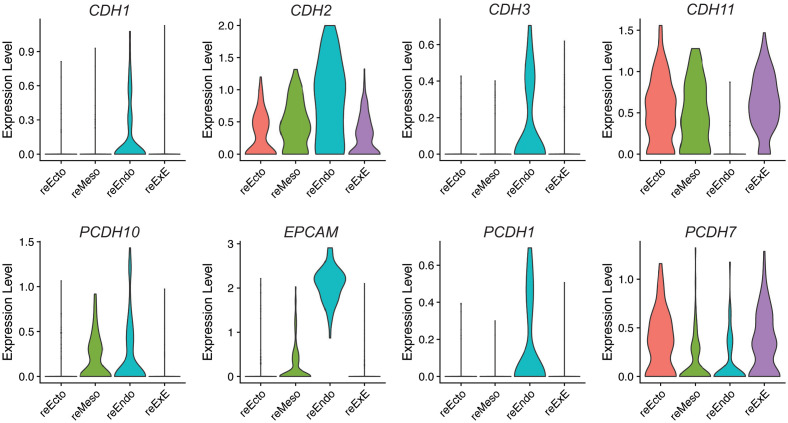
Differential expression of cell adhesion molecules in reseeded cells.

**Figure 8—figure supplement 5.**
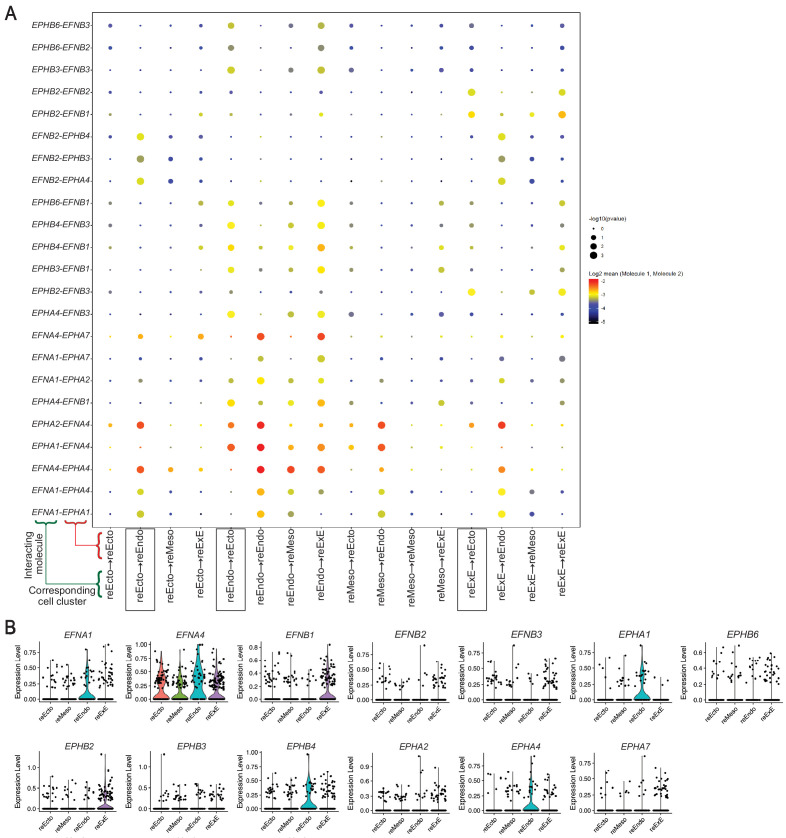
Ephrin-Eph interactions in reseeded cells. (**A**) Dot plot showing predicted Ephrin-Eph interactions between indicated pairs of cell clusters. Arrows in x-axis labels indicate direction of signaling (signaling to receiving cluster). In each interacting pair in y-axis, first partner is expressed in the signaling cluster and second partner is expressed in the receiving cluster; *p* value indicates enrichment of predicted Ephrin-Eph interactions among all predicted receptor-ligand interactions (see Source Data File 7); mean indicates average expression of receptor and ligand in interacting cluster. (**B**) Violin plot illustrating expression of genes encoding Ephrins and Ephs from the interaction analysis.

While cell sorting experiments have been performed in frog and fish gastrulae comprised of the three germ layers (Davis et al., 1997; Klopper et al., 2010; Ninomiya and Winklbauer, 2008), it is unclear whether ExE cells sort relative to embryonic cells. In our assay, CDX2^+^ ExE-like cells readily mixed with T^+^ and SOX17^+^ cells, but tended to segregate from SOX2^+^ cells, as shown by immunofluorescence images (Figure 8A–B, Figure 8—figure supplement 1A) and significantly higher cosine value between expression domains of T or SOX17 and CDX2 compared to that between SOX2 and CDX2 across all densities at all time-points (p<0.0001; Figure 8C, Figure 8—figure supplement 2). Since SOX17 expression was shared by Endoderm and hPGCLC, we used FOXA2 and GATA6 to identify definitive endoderm and primitive endoderm, respectively. Similar to SOX17^+^ cells, FOXA2^+^ and GATA6^+^ cells tended to segregate from SOX2^+^ ectodermal cells (Figure 8—figure supplement 1C), but associate with GATA3^+^ ExE-like cells (Figure 8—figure supplement 1D). Taken together, these experiments reveal the ability of human germ layer and ExE-like cells from dissociated gastruloids to sort in vitro relative to different cell types, and form discrete germ layer and ExE aggregates.

### Reseeded gastruloid cells exhibit differential expression of adhesion molecules and complementary expression of Ephrin-Eph signaling components

In cell sorting experiments, we used immunofluorescence staining of SOX2, T, SOX17, and CDX2 to identify ectoderm, mesoderm, endoderm, and ExE-like cells, respectively. To correlate sorting behaviors observed in these studies with scRNA-seq data, we combined clusters with strong expression of *SOX2* (EPI-like and Ectoderm), *T* (Mesoderm-1 and -2), *SOX17* (Endoderm and hPGCLC), and *CDX2* (ExE-like), termed reEcto, reMeso, reEndo, and reExE, respectively (Figure 8—figure supplement 3).

We first asked whether differential adhesion could be responsible for aggregation of same cell types by investigating expression of cell adhesion components. Interestingly, *SOX17^+^* reEndo cluster exhibited the highest expression of genes encoding classical cadherins that promote cell adhesion via homophilic and heterophilic interactions (*CDH1*, *CDH2*, *CDH3*), protocadherin *PCDH1*, and *EPCAM* (>2 fold over other clusters) (Takeichi, 1990). *PCDH10* expression was enriched in reEndo and reMeso clusters (>2 fold over other clusters). In contrast, *CDH11* was enriched in reEcto, reMeso, and reExE clusters (>2 fold over reEndo cluster), while *PCDH7* was enriched in reEcto and reExE clusters (>2 fold over other clusters) (Figure 8—figure supplement 4).

Ephrin/Eph signaling has been shown to underlie cell sorting or tissue separation in germ layers of *Xenopus* gastrulae (Fagotto et al., 2013; Rohani et al., 2011). We used CellPhoneDB (Efremova et al., 2020), which predicts ligand-receptor interactions between pairs of cell types using published datasets. Based on gene expression, CellPhoneDB predicted multiple Ephrin/Eph interacting pairs (Figure 8—figure supplement 5), implying a potential role of the Ephrin/Eph pathway in the selective cell sorting behaviors of reseeded gastruloid cells. Overall, our experiments revealed that gastruloid cells, when dissociated and reseeded, exhibit cell sorting behavior. Future studies will test whether differential expression of cell adhesion and complementary expression of Ephrin/Eph molecules underlie the formation of discrete aggregates by reseeded gastruloid cells.

## Discussion

**Figure 9.**
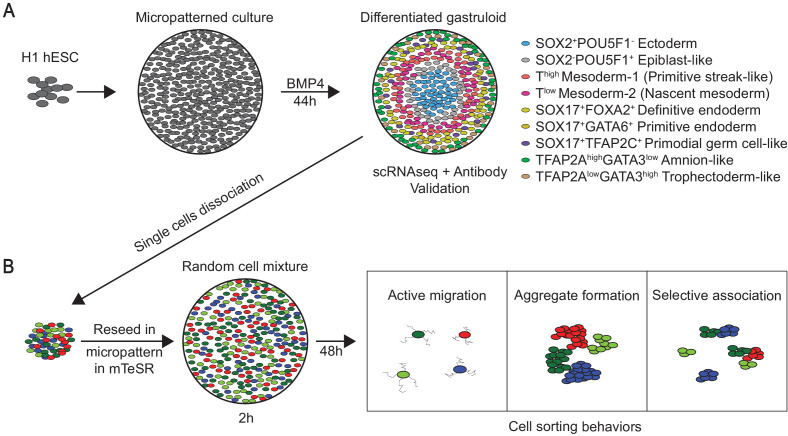
BMP4-differentiated gastruloids generate primate-relevant gastrulation cell types that show cell sorting behaviors when dissociated and reseeded in single-cell mixture. (**A**) Immunofluorescence and scRNA-seq analyses identify seven major cell types relevant during primate gastrulation. (**B**) Gastruloids dissociated into single cells and reseeded onto micropatterns show cell sorting behaviors.

Human gastrulation remains unstudied due to ethical and experimental limitations. However, forward and reverse genetics studies in research animals provide deep insights into germ layer induction, patterning, and morphogenesis that inform hESC-based experimental platforms to investigate aspects of human gastrulation (Rossant and Tam, 2017; Shahbazi and Zernicka-Goetz, 2018; Simunovic and Brivanlou, 2017; Taniguchi et al., 2019). This work adopts and extends the 2D micropatterned gastruloid system established by Brivanlou and collaborators (Warmflash et al., 2014). Our results corroborate that BMP4 treatment of hESCs on ECM micro-discs reproducibly generates radially-arranged cellular rings that resemble the three germ layers and ExE cells (Warmflash et al., 2014). Unbiased computational analyses of the scRNA-seq data from 72 gastruloids (2475 cells expressing 23,271 genes) reveal a higher cellular complexity of gastruloids that encompasses seven cell types: EPI-like, Ectoderm, Mesoderm-1, –2, Endoderm, hPGCLC, and ExE-like, and their transcriptomes. Using canonical markers, cross-species analyses, and comparison with datasets from hESC-differentiated cell types, we report characteristics previously undescribed in hESC gastruloids, such as the presence of transitional cell state in mesoderm, primitive and definitive endoderm signatures, as well as primate-specific hPGCLC and ExE-like cells with TE and amnion signatures (Figure 9A). Furthermore, we show that upon gastruloid dissociation, the resulting single cells exhibited cell sorting capabilities when reseeded in vitro (Figure 9B), similar to germ layer cells in frog and fish embryos.

During embryogenesis, gene expression is dynamic along the developmental timeline (Niu et al., 2019). Defining the developmental stage of the hESC gastruloid is crucial for determining cell types generated and its ability to model human gastrulation. We showed that gastruloids correspond closest to E7.0 mouse (Pijuan-Sala et al., 2019) and 16 dpf cyno monkey (Ma et al., 2019) gastrulae based on predicted cell stage scores, gene expression correlation, and predicted cell type composition (Figure 3E–H, Figure 3—figure supplement 1). Moreover, the majority of gastruloid cells was predicted to correspond to E7.0–7.5 in mouse (92%) and 14–17 dpf in monkey (83%; Figure 3C–D). Together with the prominent representation of post-implantation epiblast and PS-like cell types in the gastruloids (Figure 3I–J), these analyses suggest that hESC gastruloids can model early-mid stages of human gastrulation, likely corresponding to 6–7 Carnegie stages of human embryos (O’Rahilly and Müller, 1987).

Toward better understanding of monkey gastrulating cell types (Nakamura et al., 2016), which are not as well-defined as in mouse (Peng et al., 2019), we performed interspecies comparison using published datasets (Ma et al., 2019; Pijuan-Sala et al., 2019; Figure 3—figure supplement 3). Few monkey cells were predicted to correspond to mouse ectodermal derivatives or definitive endoderm, suggesting that the three germ layers have not fully developed by 17 dpf in the in vitro cultured monkey embryos. Nonetheless, we found similarities between epiblast cell types, gastrulating cells (E-Gast and L-Gast1/2) to mouse PS and mesoderm, VE/YE to mouse ExE Endoderm, and TE to mouse ExE Ectoderm, suggesting conservation in the formation of these cell types. However, monkey E-PGCs were primarily predicted to correspond to mouse epiblast, PS, and nascent mesoderm than PGC, reflecting species differences in PGC formation (Tan and Tee, 2019).

Interspecies comparison with hESC gastruloids allowed us to compare gastruloid clusters identified through canonical markers with mouse and cyno monkey gastrulae cell types based on transcriptomes. We identified EPI-like cluster resembling post-implantation epiblast in mouse, monkey, and human (Figures 3I–J and 4A), and Ectoderm cluster expressing both nonneural and neural ectoderm markers enriched in mouse gastrulae (Pijuan-Sala et al., 2019; Figure 4D). The Endoderm cluster resembles both primitive and definitive endoderm (Figure 3I–J), but the distinction is unclear (Figure 4—figure supplement 6), likely due to the transcriptional similarity between these two cell types, as reported in mouse (Nowotschin et al., 2019). The gastruloid Mesoderm-1 and -2 resemble mouse PS and nascent mesoderm, and monkey gastrulating cells (Figure 3I–J). In monkey, two late gastrulating populations were reported, L-Gast1 and L-Gast2, enriched in gastrulating signatures and mesoderm development signatures, respectively (Ma et al., 2019). Indeed, interspecies comparison showed higher proportion of monkey cells in L-Gast1 and L-Gast2 corresponding to mouse PS and nascent mesoderm, respectively (Figure 3—figure supplement 3C). Likewise, higher proportion of gastruloid Mesoderm-1 and -2 cells were predicted to correspond to monkey L-Gast1 and L-Gast2, respectively (Figure 3J). Moreover, pseudotime placed Mesoderm-2 after Mesoderm-1 (Figure 4B), suggesting that hESC gastruloids model conserved aspects of mesoderm formation. Demonstrating that this system can also uncover primate-specific molecular mechanisms, we found a shared *FGF8^low^FGF17^high^* signature in gastruloid mesodermal and monkey gastrulating cells, but not in mouse (Figure 4—figure supplement 3D), where FGF8 is required for cell migration during gastrulation (Sun et al., 1999). As more scRNA-seq datasets and tools emerge, similarities and differences between human and animal gastrulation can be further investigated.

Early gastrulation stages are marked by the formation of PS, and in its anterior region, of the ‘organizer’, a conserved signaling center which contributes to axis induction and germ layer patterning in vertebrates (De Robertis et al., 2000; Spemann and Mangold, 2001). Organizer formation and its functional competence has been reported in micropatterned differentiation using WNT and ACTIVIN but not with BMP4 (Martyn et al., 2018). Accordingly, we have not identified organizer or axial mesoderm cell types in the BMP4-induced 2D gastruloids.

The gastruloid hPGCLC cluster, enriched in human/primate PGC markers, *NANOS3*, *SOX17*, *TFAP2C, PRDM1,* and *NANOG* (Chen et al., 2019; Sasaki et al., 2016), showed strong correspondence to monkey PGCs (Figure 3J), and hESC-derived hPGCLCs in 2D (Chen et al., 2019) and 3D (Zheng et al., 2019) culture conditions (Figure 5D). However, we did not find similarities with mouse PGCs, consistent with differences in PGC formation between mouse and human (Tan and Tee, 2019). Additionally, CDX2^+^ gastruloid cells were first reported as presumptive TE (Warmflash et al., 2014), but recently suggested to instead resemble mouse ExE mesoderm (Martyn et al., 2019b). Our analysis indicated transcriptional similarity of these ExE-like cells to TE and amnion of in vitro cultured monkey and human embryos (Figures 3J and 6D), as well as mouse ExE ectoderm, a derivative of polar TE, but not to mouse ExE mesoderm (Figure 3I), suggesting that micropatterned gastruloid platform captures species-specific features in the formation of TE-like cells. ScRNA-seq showed co-expression of TE (*CDX2*, *GATA3*, *KRT7*) and amnion (*TFAP2A*, *WNT6*, *ISL1*) markers, although immunofluorescence studies revealed some heterogeneity in the ExE-like population as indicated by the presence of GATA3^high^TFAP2A^low^ and GATA3^low^TFAP2A^high^ cells (Figure 6B and C). We interpret these data to indicate that the outermost gastruloid ring contains precursors of TE-like and amnion-like cells, is a mix of both cell types, or represents a cell type with mixed characteristics that does not exist in vivo.

Notably, BMP4 treatment of hESCs within a short period of time (2 to 4 days) results in diverse cell fates in 2D micropatterns (Figure 2), disorganized 3D aggregates (Chen et al., 2019), or controlled 3D microfluidic cultures (Zheng et al., 2019). It is particularly intriguing that BMP4 co-induces hPGCLC and amnion-like cells in these in vitro systems, given that current cyno monkey studies posit induction of PGCs in amnion by BMP4, along with WNT3A (Sasaki et al., 2016). Since some SOX17^+^TFAP2C^+^ gastruloid hPGCLCs co-expressed amnion marker TFAP2A at protein and RNA levels (Figure 5—figure supplement 1D–E), it will be of interest to test whether hPGCLCs arise from the amnion-like ExE cells in the gastruloid system. Highlighting the conserved role of BMP4 in PGC induction in mammals, BMP4 signaling from ExE ectoderm to epiblast cells was recognized as an instructive signal toward PGC fates in mouse (Lawson et al., 1999). Interestingly, however, the micropatterned differentiation of mouse pluripotent stem cells did not appear to form PGC-like cells (Morgani et al., 2018). Future comparisons of micropatterned cultures between human and mouse pluripotent stem cells should facilitate comparative studies of signaling responses and gene regulatory networks between species in controlled and comparable culture conditions (Chhabra et al., 2019; Etoc et al., 2016; Morgani et al., 2018).

Cell sorting behaviors have been classically described in frog and fish embryos, where single cells from dissociated gastrulae were allowed to interact in suspension in vitro (Krens and Heisenberg, 2011; Townes and Holtfreter, 1955). Mouse and human ESCs have remarkable self-organization capacity into blastocyst-like structures (Shahbazi et al., 2019). However, it is unknown whether mammalian, including human germ layer and ExE cells, can undergo cell sorting in vitro. To this end, we showed that human gastruloids, when dissociated and reseeded as single cells onto the ECM discs, exhibited cell sorting behaviors reported for amphibians and fish such as active migration, homogeneous aggregate formation, and selective association with and/or avoidance of distinct cell types (Figure 9B). Gastruloid ectodermal cells associated more readily with mesodermal cells than endodermal cells, while ExE-like CDX2^+^ cells tended to associate with mesendodermal cells but segregate from ectodermal cells. However, we did not observe an engulfment of ectoderm by mesoderm or endoderm, as reported in 3D aggregates or tissue explant cultures (Krieg et al., 2008; Schötz et al., 2008), likely explained by 2D conditions in our sorting assay. Overall, our results indicate that human gastruloid cells have the ability to undergo sorting, motivating future exploration of their self-organization potential.

Three main models – differential adhesion, differential cortical tension, and contact inhibition – have been proposed to explain cell sorting (Fagotto, 2015). Our gene expression analysis suggests that cadherins may be involved in the sorting of dissociated gastruloid cells through homophilic and/or heterophilic adhesion, as indicated by the expression of specific cadherins in different cell types (Figure 8—figure supplement 4). Complementary expression of Ephrins and Eph receptors in the segregating cell clusters suggested the involvement of contact inhibition cues, which underlie complex functional interactions across tissues during animal embryogenesis (Fagotto, 2015; Fagotto et al., 2014).

Our work provides a rich resource for transcriptomic signatures of human germ layers and ExE cell types at early-mid gastrula stage, differentiated in vitro in a stereotyped arrangement. This resource can be applied in the studies of the cellular and molecular mechanisms of human gastrulation, and can be used to identify candidate markers for different cell types in human gastrulae. This BMP4-induced 2D gastruloid system lacks some cell types found in mouse gastrula, such as axial mesoderm or ExE mesoderm. However, we found amnion- and PGC-like cells, which are transcriptionally similar to cyno monkey gastrulae (Ma et al., 2019). Thus, our work underscores the utility of this 2D micropatterned hESC culture to investigate aspects of human development (Siggia and Warmflash, 2018; Simunovic and Brivanlou, 2017), but also the significance of studying human and nonhuman primate embryos in vivo and cultured in vitro (Deglincerti et al., 2016; Ma et al., 2019; Nakamura et al., 2016; Niu et al., 2019; Shahbazi et al., 2016; Xiang et al., 2020) to provide a critical reference for interpreting in vitro embryogenesis models. Future studies involving gene expression profiles over the course of gastruloid formation will allow mapping and comparison of the human developmental timeline and dynamic gene regulatory networks with animal gastrulae. Such studies will help delineate the similarities and differences between molecular mechanisms and signaling pathways underlying gastrulation in human and animal models, and in generating experimental platforms for understanding the pathologies of human embryogenesis.

## Materials and methods

**Key resources table:** 

Reagent type (species) or resource	Designation	Source or reference	Identifiers	Additional information
Cell line (Human)	WA01 (H1) human embryonic stem cell	WiCell	RRID:CVCL_9771, hPSCReg ID: WAe001-A	
Cell line (Human)	WA09 (H9) human embryonic stem cell	WiCell	RRID:CVCL_9773, hPSCReg ID: WAe009-A	
Antibody	anti-SOX2 (Mouse monoclonal)	Cell Signaling Technology	Cat# 4900, RRID:AB_10560516	(1:200)
Antibody	anti-SOX2 (Rabbit monoclonal)	Cell Signaling Technology	Cat# 3579, RRID:AB_2195767	(1:200)
Antibody	anti-SOX1 (Goat polyclonal)	R and D Systems	Cat# AF3369, RRID:AB_2239879	(10 μg/mL)
Antibody	anti-T (Goat polyclonal)	R and D Systems	Cat# AF2085, RRID:AB_2200235	(2 μg/mL)
Antibody	anti-SOX17 (Goat polyclonal)	R and D Systems	Cat# AF1924, RRID:AB_355060	(1 μg/mL)
Antibody	anti-CDX2 (Rabbit monoclonal)	Cell Signaling Technology	Cat# 12306, RRID:AB_2797879	(1:100)
Antibody	anti-pSMAD1 (Rabbit monoclonal)	Cell Signaling Technology	Cat# 9516, RRID:AB_491015	(1:200)
Antibody	anti-CDH1 (Rabbit monoclonal)	Cell Signaling Technology	Cat# 3195, RRID:AB_2291471	(1:400)
Antibody	anti-CDH2 (Mouse monoclonal)	Cell Signaling Technology	Cat# 14215, RRID:AB_2798427	(1:800)
antibody	anti-GATA3 (Rabbit monoclonal)	Cell Signaling Technology	Cat# 5852, RRID:AB_10835690	(1:1600)
Antibody	anti-GATA3 (Mouse monoclonal)	R and D Systems	Cat# MAB6330, RRID:AB_10640512	(1:500)
Antibody	anti-GATA6 (Rabbit monoclonal)	Cell Signaling Technology	Cat# 5851, RRID:AB_10705521	(1:1600)
Antibody	anti-FOXA2 (Rabbit monoclonal)	Cell Signaling Technology	Cat# 8186, RRID:AB_10891055	(1:800)
Antibody	anti-OCT3/4(POU5F1) (Mouse monoclonal)	Santa Cruz Biotechnology	Cat# sc-5279, RRID:AB_628051	(1:200)
Antibody	anti-ZO-1 (Mouse monoclonal)	Thermo Fisher Scientific	Cat# 33–9100, RRID:AB_2533147	(1:500)
Antibody	anti-KRT7 (Rabbit monoclonal)	Cell Signaling Technology	Cat# 4465, RRID:AB_11178382	(1:200)
Antibody	anti-AP-2α (Rabbit monoclonal)	Santa Cruz Biotechnology	Cat# sc-12726, RRID:AB_667767	(1:200)
Antibody	anti-AP-2γ (Rabbit polyclonal)	Cell Signaling Technology	Cat# 2320, RRID:AB_2202287	(1:800)
Chemical compound, drug	Gentle Cell Dissociation Reagent	Stemcell Technologies	Cat # 07174	
Chemical compound, drug	Accutase solution	Sigma Aldrich	Cat # A6964	
Chemical compound, drug	RPMI 1640 Medium	Thermo Fisher Scientific	Cat # 11879020	
Chemical compound, drug	ROCK Inhibitor (Y-27632)	Millipore Sigma	Cat # SCM075	(10 μM)
Chemical compound, drug	Recombinant Human BMP-4 Protein	R and D Systems	Cat # 314 BP	(50 ng/mL)
Chemical compound, drug	mTeSR1	Stemcell Technologies	Cat # 85857	
Commercial assay, kit	Chromium Single Cell 3’ Library and Gel Bead Kit v2	10xGenomics	Cat # 120237	
Commercial assay, kit	Chromium Single Cell 3’ Chip Kit v2	10xGenomics	Cat # 120236	
Commercial assay, kit	Chromium i7 Multiplex Kit	10xGenomics	Cat # 120262	
Software, algorithm	Seurat 3.1	Stuart et al., 2019 Butler et al., 2018	Seurat, RRID:SCR_016341	scRNA-seq analysis
Software, algorithm	CellPhoneDB	Efremova et al., 2020		Receptor-ligand interaction analysis
Software, algorithm	R Bioconductor *destiny*	Angerer et al., 2016 (https://bioconductor.org/packages/release/bioc/html/destiny.html)		Pseudotime and transition probabilities
Software, algorithm	ImageJ/FIJI	ImageJ/FIJI	RRID:SCR_002285	Image analysis
Software, algorithm	FIJI plugin – Concentric Circles	https://imagej.nih.gov/ij/plugins/concentric-circles.html		Image analysis
Software, algorithm	FIJI plugin – ClearVolume	Royer et al., 2015 (https://imagej.net/ClearVolume)		Image analysis
Software, algorithm	Prism 8	Graphpad	RRID:SCR_002798	Statistics and graphs
Other	DAPI	Millipore Sigma	Cat# D9542	(0.4 μg/mL)
Other	Donkey anti-Mouse IgG (H+L) Highly Cross-Adsorbed Secondary Antibody, Alexa Fluor 488	Thermo Fisher Scientific	Cat# A-21202, RRID:AB_141607	(1:500)
Other	Donkey anti-Goat IgG (H+L) Cross-Adsorbed Secondary Antibody, Alexa Fluor 555	Thermo Fisher Scientific	Cat# A-21432, RRID:AB_2535853	(1:500)
Other	Donkey anti-Rabbit IgG (H+L) Highly Cross-Adsorbed Secondary Antibody, Alexa Fluor 647	Thermo Fisher Scientific	Cat# A-31573, RRID:AB_2536183	(1:500)

### Cell line verification and testing

Cell lines used in this study have been authenticated using Short Tandem Repeat (STR) profiling, and are regularly tested and negative for mycoplasma contamination.

### PDMS stamp fabrication

Standard photolithography methods were used to fabricate the Master, which was used as a template in molding stamps for micro-contactprinting (Alom Ruiz and Chen, 2007; Théry and Piel, 2009). Briefly, the silicon wafer was cleaned by dipping in hydrogen fluoride solution for 1 min and rinsing with water twice. A thin layer of photoresist, SU-8 3000 (Kayaku Advanced Materials, Westborough, MA) was then spin-coated on the wafer at 500 rpm for 10 s, followed by 3,000 rpm for 30 s. The wafer with spin-coated photoresist layer was baked at 65°C for 5 min and 95°C for 15 min. The optical mask with desired features was placed in contact with photoresist layer and illuminated with the UV light at 150–250 mJ/cm^2^ at 350–400 nm. The photoresist layer was then baked at 65°C for 5 min and 95°C for 10 min. Subsequently, the photoresist was developed in developer solution for 5 min. The photoresist Master was then coated with a layer of chlorotrimethylsilane in the vacuum for 30 min. Polydimethylsiloxane (PDMS) and its curing agent, Sylgard 184, (Dow Corning, Midland, MI) in 10:1 ratio were mixed, degassed, poured over the top of Master, and cured at 60°C overnight, after which the PDMS layer was peeled off to be used as a stamp in micro-contactprinting.

### Microcontact printing

PDMS stamps (approximately 1 x 1 cm) were sterilized by washing in ethanol solution and dried in a laminar flow hood. The stamps were treated with O_2_ plasma at 100–150 microns for 2 min. Growth factor reduced Matrigel (Corning, Corning, NY) was diluted in DMEM/F-12 (Gibco, Waltham, MA) at 1:20 dilution and incubated on the stamps to cover the entire surface of the feature side at room temperature for 45 min. Matrigel solution was then aspirated off the stamps, which were air-dried. Using tweezers, Matrigel-coated surface of stamps were brought in contact with glass or plastic substrate for 2 min, ensuring conformal contact between features and substrate. The stamps were then removed and rinsed in ethanol for future uses. Matrigel-printed substrates were incubated with 0.1% Pluronic F-68 (Gibco) in DPBS-/-at room temperature for 1 hr. In some experiments, we skipped incubation with Pluronic F-68, and did not observe a difference in cell attachment. Finally, the substrates were washed with DPBS-/-for four times. Matrigel-printed substrates were stored in DPBS-/-solution at 4°C for up to 2 weeks.

### Cell seeding protocol

Two human embryonic stem cell lines were used in this study: H1 and H9 (WiCell, Madison, WI). Both cell lines were routinely cultured on six-well plates coated with growth factor reduced Matrigel in mTeSR media (Stemcell Technologies, Vancouver, Canada) with daily media replacement. The cells were passaged at 80% confluence using Gentle Cell Dissociation Reagent (Stemcell Technologies) per manufacturer’s protocol. Both cell lines were cultured at 37°C and 5% CO_2_.

For gastruloid differentiation, we adapted the protocol developed by Warmflash et al., 2014. H1 and H9 cells at 80% confluence were collected by Accutase (Sigma Aldrich, St. Louis, MO) incubation at 37°C and 5% CO_2_ for 8 min, after which equal volume of RPMI medium 1640 (Life Technologies, Carlsbad, CA) was added. The cell solution was centrifuged at 300 rcf (relative centrifugal force) for 5 min, after which the supernatant was removed. A single-cell suspension was then generated with fresh mTeSR. Cells were counted using Countess II Automated Cell Counter (Life Technologies) per manufacturer's instructions and seeded onto the micropattern at 132,000–263,000 cells/cm^2^ in mTeSR with 10 μM Rho-associated kinase inhibitor (ROCKi Y-27632, Millipore Sigma, Burlington, MA). After 6 hr, medium containing ROCKi was replaced with mTeSR. The cells were cultured at this stage for 2 hr, after which the medium was replaced with mTeSR containing 50 ng/mL BMP4 (R and D Systems, Minneapolis, MN) for 42–48 hr.

For cell sorting experiments, gastruloids that have been treated with BMP4 for 44 hr were collected by Accutase incubation at 37°C and 5% CO_2_ for 10 min, after which an equal volume of RPMI medium 1640 was added. A single-cell suspension was generated by gently pipetting the cell suspension for five times. The cell suspension was centrifuged at 300 rcf for 5 min, after which the supernatant was removed. A single-cell suspension was then generated with fresh mTeSR. Cells were then counted and seeded onto fresh micropattern at 72,000 cells/cm^2^, 143,000 cells/cm^2^, or 286,000 cells/cm^2^ in mTeSR with 10 μM ROCKi Y-27632. After 4 hr, medium containing ROCKi was replaced with mTeSR. mTeSR was replaced daily to wash away unattached cells.

### Immunocytochemistry

Cells were rinsed once with PBS, fixed in 4% paraformaldehyde for 30 min, and rinsed twice with PBS at room temperature. Blocking solution was made with 0.1% Triton-X and 3% Normal Donkey Serum (Jackson Immunoresearch, West Grove, PA) in PBS and washing solution was made with 0.1% Tween-20 in PBS. Fixed cells were incubated in blocking solution for 30 min at room temperature before incubation with primary antibodies in blocking solution at 4°C overnight. Cells were then washed three times in washing solution before being incubated with secondary antibodies and DAPI at 0.4 μg/mL (Invitrogen, Carlsbad, CA) in blocking solution for 1 hr. Finally, cells were washed three times in washing solution and stored in PBS or mounted with coverslips using Vectashield Antifade Mounting Medium (Vector Laboratories, Burlingame, CA).

### Microscopy and visualization of fluorescence images

All confocal images were acquired on Olympus IX81 Inverted Spinning Disk Confocal Microscope with 10X or 20X lenses. Z-stack images of ~150 µm thick were acquired in four channels corresponding to DAPI, Alexa 488, Alexa 555, and Alexa 647 conjugated antibodies. Each z-stack was projected into a single image for all channels prior to analysis. Fiji software (Schindelin et al., 2012) and its plugin ClearVolume (Royer et al., 2015) were used to visualize images.

### Quantification of fluorescence intensity

Fiji (Schindelin et al., 2012) was used to process and analyze fluorescent microscopic images. We first created masks for each fluorescent image. We then used Concentric Circles plugin to overlay 20 equally spaced concentric circles on the image of gastruloid. We measured the average fluorescence intensity along the circumference of each concentric circle. We normalized the fluorescence intensity of each marker of interest to that of DAPI, resulting in normalized average fluorescence intensity value of each marker along 20 different radii of every gastruloid colony. Finally, these values were averaged for multiple colonies per marker and presented.

### Density map-based cell counting and cosine similarity analysis

We estimated the number of cells expressing each marker in a given fluorescence image by first estimating the marker’s corresponding density map, which represents the expected spatial distribution of the number of cells over pixels in the fluorescence image acquired for the marker. Individual 2D fluorescence images were converted into 2D density maps, where the density value of a pixel is the expected number of cells at that pixel in the corresponding image. The density map of a given image *X *∈* R^NxN^* , was estimated by use of deeply-supervised fully convolutional neural network (FCNN) models (He et al., 2019) that perform an end-to-end mapping from to the desired density map, Y ∈ *R^NxN^*. The FCNN model was learned by use of a set of training data, each of which contains an annotated image and the associated ground-truth density maps. Here, each image was manually annotated by identifying the locations of centroids of cells expressing markers of interest. The ground-truth density map of each image was generated based on the annotated centroids by use of methods introduced in previous works (He et al., 2019). Specifically, 5 FCNN models, each representing SOX2, CDX2, SOX17, DAPI, and T, were separately trained and developed with corresponding annotated images. In each FCNN model, seven annotated images with 512 × 512 pixels were employed as the training images for the FCNN model training, and three annotated images were employed to validate select values of parameters in the FCNN models.

Interpreting the density map of an image as the expected spatial distribution of the number of cells over all pixels, the total number of cells in the image is calculated by summing up all the densities on the map:

$$C=\;\sum_{i=1}^{N}\sum_{j=1}^{N}Y_{ij},$$

where *Y_ij _*is the estimated density value of the location (*i,j*) in the corresponding image. Density map generation or cell counting for CDH2 and GATA3 was performed with FCNN model trained with DAPI and CDX2, respectively.

Representing the density maps corresponding to two cell distributions as *Y* ∈ *R^NxN^* and *Y'* ∈ *R^NxN^*, the spatial overlap between the two cell populations is measured based on the density maps using cosine similarity value (cosine value):

$$CosSim=\;\frac{\sum_{i=1}^{N}\sum_{j=1}^{N}Y_{ij}^{\prime }\cdot Y_{ij}}{\sqrt{\sum_{i=1}^{N}\sum_{j=1}^{N}{Y_{ij}^{\prime }}^{2}}\cdot \sqrt{\sum_{i=1}^{N}\sum_{j=1}^{N}Y_{ij}^{2}}}.$$

Cosine values ranged from 0 to 1, with one representing a complete overlap between expression patterns of two markers, whereas 0 representing no overlap. Intuitively, the cosine value based on density maps is a straightforward way to measure the spatial overlap between two cell populations (cells expressing two markers of interest). According to the definition above, pixels where both of the two cell populations have higher number of cells contribute more to the global overlap; the pixels where only one of the two or neither of them have higher densities may contribute less to the global overlap; when two cell populations have larger amount of cells, but relatively less amount of overlapped pixels, the global overlap is small.

### scRNA-seq and data analysis

Cells were collected by Accutase incubation at 37°C and 5% CO_2_ for 10 min. Cell clumps were further broken up into single cells by gently pipetting the cell solution five times, after which equal volume of RPMI medium 1640 was added. The cell suspension was centrifuged at 300 rcf for 5 min, after which the supernatant was removed. A single-cell suspension was then generated with cold DPBS-/-. Cells were then counted and resuspended at 20,000 cells per 200 μL of cold DPBS-/-in centrifuge tubes. For each cell suspension, 800 μL of cold methanol was added dropwise. The final cell suspension was incubated on ice for 15 min and kept at −80°C until use.

To prepare single-cell library, 10x Chromium Single Cell 3’ Library and Gel Bead Kit v2, Chromium Single Cell 3’ Chip Kit v2, and Chromium i7 Multiplex Kit (10X Genomics, Pleasanton, CA) were used according to the manufacturer’s instructions. cDNA libraries were then quantified on the Agilent TapeStation (Agilent Scientific Instruments, Santa Clara, CA) and sequenced on Illumina HiSeq 2500 (Illumina, San Diego, CA).

The Cell Ranger v.2.1.0 pipeline was used to align reads to the hg19.transgenes genome build and generate a digital gene expression matrix. The Seurat package (v.3.0.2) was used for further data processing and visualization. Default settings were used unless noted otherwise. For gastruloid dataset, cells with a low or high number of genes detected (Replicate 1:≤200 or ≥7,500; Replicate 2:≤200 or ≥6,000) and cells with high total mitochondrial gene expression (Replicate 1:≥3%; Replicate 2:≥2.5%) were removed from further analysis. Consequently, we selected 1,722 cells (out of 1,989) and 753 cells (out of 823) from Replicate one and Replicate 2, respectively, that passed quality control for further analysis. For 48h-reseed cultures, cells with ≤200 or ≥6000 genes detected and mitochondrial gene expression ≥2% were excluded, resulting in the selection of 479 out of 584 cells for further analysis. After normalizing and scaling the filtered expression matrix to remove unwanted sources of variation driven by mitochondrial gene expression, the number of detected UMIs, and the cell cycle, principal component analysis was performed in Seurat.

The two replicates for gastruloids were integrated using canonical correlation analysis based on ‘between-dataset anchors’ identified by Seurat, and the top 2000 highly variably expressed genes (HVGs) were included for the integrated expression matrix. Cell clusters were then identified by the Louvain community detection method using a shared nearest neighbor (SNN) resolution at 0.4 as implemented in the Seurat FindClusters function. Nonlinear dimensionality reduction by UMAP was performed on the first 15 principal components using the implementation by Seurat. We manually annotated each cluster based on known markers. Differentially expressed genes (DEG) for each cluster were identified by using FindAllMarkers with threshold settings of 0.25 log fold-change and 25% detection rate. Average gene expression correlations between two gastruloid replicates were calculated using Spearman correlation on the basis of all 23,271 genes. Gene Ontology analysis was performed using Enrichr web tool (Chen et al., 2013).

Diffusion maps for single cells were calculated based on the normalized and scaled gene expression data matrix using the R Bioconductor *destiny* package (Angerer et al., 2016), with a number of k-nearest neighbors, knn = 40, and a Gaussian kernel width, sigma = 8, slightly lower than the optimal value of sigma estimated by *destiny*. A probabilistic breadth-first search of the k-nearest neighbor graph was performed and the results of this search were converted into a pseudotime as implemented by the URD method (Farrell et al., 2018). The average cell-to-cell transition probabilities between cell types was calculated using *destiny* and presented in a heatmap.

Module score was calculated using AddModuleScore function in Seurat using default settings. This function calculates average expression of a given set of marker genes, subtracted by that of a set of randomly chosen genes (Tirosh et al., 2016).

Datasets used for comparison analyses with gastruloids include gastrulating mouse embryos (Pijuan-Sala et al., 2019), post-implantation primate cyno monkey embryos (Ma et al., 2019; Nakamura et al., 2016), in vitro cultured 6–12 dpf human embryos (Lv et al., 2019; Xiang et al., 2020), and hESC-differentiated cell types (Chen et al., 2019; Chu et al., 2016; Loh et al., 2016; Lu et al., 2018; Zheng et al., 2019). Each dataset was normalized, scaled, and processed using Seurat. Average gene expression correlations between gastruloid clusters and reference cell types were calculated using Spearman correlation on the basis of shared HVGs between gastruloids and each reference data, and presented in heatmaps. For interspecies comparison with gastrulating mouse (Pijuan-Sala et al., 2019) and post-implantation monkey embryos (Ma et al., 2019), each dataset was integrated with human gastruloid object applying Seurat anchor-based integration method. Cell type labels from mouse or monkey were transferred to human gastruloid cells using FindTranferAnchors and TransferData functions, resulting in each gastruloid cell with prediction scores for multiple mouse or monkey cell types. We defined a gastruloid cell as a predicted mouse or monkey cell type with the highest prediction score. Similar analyses were used to predict mouse or monkey cell stages in gastruloid cells. For cell type prediction analyses in interspecies comparisons, we used randomization test to identify reference cell types that map to query cell types with statistical significance of p<0.05. For randomization test for significant transferred cell types of each gastruloid cluster *i* of size *n_i_*, we sampled *n_i_* cells with replacement from all gastruloid cells 1000 times. For each of the *k^th^* time resampling of size *n_i_*, we calculated *Perc_ijk_*, the percentage of transferred cell types *j* in *n_i_* sampled cells and built a background distribution with *Perc_ij1_*, …, *Perc_ij1000_*. We then computed the p-value for cell type *j* in cluster *i* as:

$$p-value_{ij}=\frac{\#\left(Perc_{ijk}\;\geq \;the\;composition\;of\;transferred\;cell\;type\;j\;in\;cluster\;i\right)+1}{1000+1}$$

where a pseudo-count of 1 was introduced to avoid *p* values of 0. Then for cluster *i*, if *p-value_ij_* is less than or equal to 0.05, we identify transferred cell type *j* as a significant transferred cell type for cluster *i*. Similar tests were performed in monkey and mouse comparison analysis to identify mouse cell types that correspond to monkey cell types with statistical significance.

Reseed data was integrated with two replicates of gastruloids using Seurat. FindTransferAnchors and TransferData functions were used to annotate reseed cells using gastruloid data as the reference. Predicted cell type scores were calculated with Seurat, which identified nearest neighbors across gastruloid and reseed dataset, and assigned weighted score on each reseed cell based on the seven annotated gastruloid clusters (Stuart et al., 2019). CellPhoneDB (v.2.1.1) (Efremova et al., 2020), a python package, was used to investigate directionality and cross-talks of interaction between pairs of cell types.

### Statistical analysis

Randomized permutation tests were used to calculate statistical significance of cell type correspondences in interspecies comparisons. Graphpad Prism 8 software was used to perform the remaining statistical analyses: one-way ANOVA with Tukey’s multiple comparisons test, or unpaired nonparametric Mann-Whitney test, as appropriate and described in figure legends.

## Data Availability

Sequencing data have been deposited in GEO under accession code GSE144897. The following dataset was generated: MinnKTFuYCHeSDietmannSGeorgeSCAnastasioMAMorrisSASolnica-KrezelL2020High-resolution transcriptional and morphogenetic profiling of cells from micropatterned human embryonic stem cell gastruloid culturesNCBI Gene Expression OmnibusGSE14489710.7554/eLife.59445PMC772844633206048 The following previously published datasets were used: Pijuan-SalaBGriffithsJAGuibentifCHiscockTWJawaidWCalero-NietoFJMulasCIbarra-SoriaXTyserRCVHoDLLReikWSrinivasSSimonsBDNicholsJMarioniJCGöttgensB2019Timecourse single-cell RNAseq of whole mouse embryos harvested between days 6.5 and 8.5 of developmentArrayExpressE-MTAB-6967 MaHZhaiJWanHJiangXWangXWangLXiangYHeXZhaoZ-AZhaoBZhengPLiLWangH2019In vitro culture of cynomolgus monkey embryos beyond gastrulationNCBI Gene Expression OmnibusGSE13011410.1126/science.aax789031672918 NakamuraTOkamotoISasakiKYabutaYIwataniCTsuchiyaHSeitaYNakamuraSYamamotoTSaitouM2016A developmental coordinate of the spectrum of pluripotency among mice, monkeys, and humansNCBI Gene Expression OmnibusGSE7476710.1038/nature1909627556940 LvBAnQZengQZhangXLuPWangYZhuXJiYFanGXueZ2019Single-cell RNA sequencing reveals regulatory mechanism for trophoblast cell-fate divergence in human peri-implantation embryoNCBI Gene Expression OmnibusGSE12561610.1371/journal.pbio.3000187PMC680285231596842 XiangLYinYZhengYMaYLiYZhaoZGuoJAiZNiuYDuanKHeJRenSWuDBaiYShangZDaiXJiWLiT2019A developmental landscape of 3D-cultured human pre-gastrulation embryosNCBI Gene Expression OmnibusGSE13644710.1038/s41586-019-1875-y31830756 ChuL-FLengNZhangJHouZMamottDVereideDTChoiJKendziorskiCStewartRThomsonJA2016Snapshot and temporal scRNA-seq of progenitor cells to dissect human embryonic stem cells entry into endoderm progenitorsNCBI Gene Expression OmnibusGSE75748 LohKMChenAKohPWDengTZSinhaRTsaiJMBarkalAAShenKYJainRMorgantiRMShyh-ChangNFernhoffNBGeorgeBMWernigGSalomonREAChenZVogelHEpsteinJAKundajeATalbotWSBeachyPAAngLTWeissmanIL2016In vitro cultured H7 human embryonic stem cells (WiCell) and H7-derived downstream early mesoderm progenitorsNCBI Gene Expression OmnibusGSE85066 LuJBacceiALummertz da RochaEGuillermierCMcManusSFinneyLAZhangCSteinhauserMLLiHLerouPH2018Single-Cell RNA Sequencing Reveals Metallothionein Heterogeneity during hESC Differentiation to Definitive EndodermNCBI Gene Expression OmnibusGSE10997910.1016/j.scr.2018.01.01529427839 ChenDSunNHouLKimRFaithJAslanyanMTaoYZhengYFuJLiuWKellisMClarkA2019Human Primordial Germ Cells are Specified from Lineage Primed ProgenitorsNCBI Gene Expression OmnibusGSE14002110.1016/j.celrep.2019.11.083PMC693967731875561 ZhengYXueXShaoYWangSEsfahaniSNLiZMuncieJMLakinsJNWeaverVMGumucioDLFuJ2019Single-cell RNA-Sequencing data of posteriorized embryonic-like sacs, amniotic ectoderm-like cells, and H9 hESCNCBI Gene Expression OmnibusGSE134571

## Additional files

Source data 1. Metadata for gastruloid cells including cluster annotation, replicate number, and predicted mouse and monkey cell types and stages. Average gene expression correlation values between replicates 1 and 2.

Source data 2. Differentially expressed genes of gastruloid clusters.

Source data 3. Shared highly variable genes (HVGs) between gastruloid and reference datasets used for average gene expression correlation, and correlation values.

Source data 4. Metadata for cyno monkey cells including monkey cell type and developmental stage, and predicted mouse cell type and developmental stage. Shared HVGs between mouse and monkey dataset, and gene expression correlation values of developmental stages.

Source data 5. Metadata for reseed cells including cluster annotation, and prediction scores for gastruloid cell types.

Source data 6. Downregulated and upregulated genes, shared HVGs, and gene expression correlation values between reseed and gastruloids.

Source data 7. Predicted interactions between reseed clusters.

Transparent reporting form
