# Supplementary material for: High-resolution transcriptional and morphogenetic profiling of cells from micropatterned human ESC gastruloid cultures
Source: eLife. 2020 Nov 18;9:e59445. doi: 10.7554/eLife.59445 (PMC7728446; doi:10.7554/eLife.59445)
Supplement: Peer review history

# Decision letter

**DOI:** 10.7554/eLife.59445.sa1

**Affiliations:** University of Wisconsin, MadisonUnited States

In the interests of transparency, eLife publishes the most substantive revision requests and the accompanying author responses.

**Acceptance summary:**

The manuscript includes a combination of scRNA-seq and classical cell sorting experiments to a 2D human gastruloid model. By comparing with data from other species, the data show that the model represents the early to mid-gastrula and that 2 subtypes of mesoderm as well as germ cells, can be identified, revealing more complexity in the model than has been previously shown. The cell sorting experiments reveal that human cells have cell sorting properties similar to those of amphibian cells in classical experiments. The manuscript provides a number of important insights as well as a valuable dataset.

**Decision letter after peer review:**

Thank you for submitting your article "High-resolution transcriptional and morphogenetic profiling of cells from micropatterned human ESC gastruloid cultures" for consideration by *eLife*. Your article has been reviewed by three peer reviewers, and the evaluation has been overseen by a Reviewing Editor and Michael Eisen as the Senior Editor. The following individuals involved in review of your submission have agreed to reveal their identity: Aryeh Warmflash (Reviewer #1); Kat Hadjantonakis (Reviewer #3).

The reviewers have discussed the reviews with one another and the Reviewing Editor has drafted this decision to help you prepare a revised submission.

We would like to draw your attention to changes in our revision policy that we have made in response to COVID-19 (https://elifesciences.org/articles/57162). Specifically, we are asking editors to accept without delay manuscripts, like yours, that they judge can stand as *eLife* papers without additional data, even if they feel that they would make the manuscript stronger. Thus the revisions requested below only address clarity and presentation.

Summary:

In this manuscript, the authors apply scRNA-seq to a 2D human gastruloid model. They also perform classical cell sorting experiments using the cells differentiated in this gastruloid model. The scRNA-Seq data will provide a very useful resource to the community studying gastrulation. It also provides some novel insights regarding the gastruloid model. By comparing with data from other species, the authors show that the model represents the early to mid-gastrula. The authors also identify 2 subtypes of mesoderm as well as germ cells, revealing more complexity in the model than has been previously shown. The cell sorting experiments reveal that human cells have cell sorting properties similar to those that have been shown for amphibian cells in classical experiments. Altogether, the manuscript provides a number of important insights as well as a valuable dataset. It is clearly written and the conclusions are supported by data.

Essential revisions:

1) Additional clarification of statistical analysis is needed. Some of the differences in the cell type, interspecies comparisons and interaction between selected receptor-ligand pair are relatively small and so inclusion of confidence intervals (or additional details of analysis) would confirm that these differences are robust.

2) In some cases, it isn't clear whether there are mixed populations of cells or cells with mixed characteristics. The authors should attempt to distinguish between these two possibilities by assessing the scRNAseq data to determine if the cells can be split into two groups or whether markers of the two lineages are co-expressed. This should be carried out for 1) amnion and trophoectoderm , 2) in the ectodermal cluster -neural cells versus surface ectoderm and 3) for primitive and definitive endoderm.

3) The identity of large numbers of cells as ExM in the gastruloid model is confusing. The authors comment on this in the Discussion and note that it may be biased by the large proportion of ExM cells in the monkey dataset. It would be useful to include this caveat where the data is originally discussed to avoid giving misleading impressions.

# Reply: Author response

**DOI:** 10.7554/eLife.59445.sa2

Essential revisions:

1) Additional clarification of statistical analysis is needed. Some of the differences in the cell type, interspecies comparisons and interaction between selected receptor-ligand pair are relatively small and so inclusion of confidence intervals (or additional details of analysis) would confirm that these differences are robust.

To test statistical significance in interspecies comparisons, we implemented a randomization test to identify mouse or monkey cell types that map to a gastruloid cell type with statistical significance of *p < 0.05*. The results of this statistical analyses are incorporated in Figure 3I and J, and the implementation method is explained in the Materials and methods section.

The enrichment of predicted Eph-ephrin pairs (*p value*) shown in Figure 8—figure supplement 5, is based on the comparison against all receptor-ligand interaction. No statistical test was performed specifically among Eph-ephrin interactions. We rephrased the text to emphasize Eph-ephrin complementary expression in distinct cell types but not enrichment between pairs of cell types.

2) In some cases, it isn't clear whether there are mixed populations of cells or cells with mixed characteristics. The authors should attempt to distinguish between these two possibilities by assessing the scRNAseq data to determine if the cells can be split into two groups or whether markers of the two lineages are co-expressed. This should be carried out for 1) amnion and trophoectoderm , 2) in the ectodermal cluster -neural cells versus surface ectoderm and 3) for primitive and definitive endoderm.

We have carried out additional analyses, but they could not separate sub cell types within the three cell populations. We updated our figures to show expression of additional cell type-specific markers: neural versus surface markers in Ectoderm cluster (Figure 4—figure supplement 2), primitive versus definitive endoderm markers in Endoderm cluster (Figure 4—figure supplement 6), and amnion versus trophectoderm markers (updated Figure 6C). These analyses support the notion that ExE cells co-express markers of trophectoderm and amnion, and cells in Endoderm cluster express markers of definitive endoderm and primitive endoderm. We also discuss that the lack of cells with clear trophectoderm and primitive endoderm identities in the 2D gastruloid culture is consistent with H1 (or H9) conventional/primed hESCs resembling post-implantation epiblast. Indeed, primed hESCs have not been convincingly differentiated into pre-implantation trophoblast or primitive endoderm lineages.

3) The identity of large numbers of cells as ExM in the gastruloid model is confusing. The authors comment on this in the Discussion and note that it may be biased by the large proportion of ExM cells in the monkey dataset. It would be useful to include this caveat where the data is originally discussed to avoid giving misleading impressions.

In agreement, we have included this caveat in the Results. As illustrated in Figure 4—figure supplement 4, top 50 monkey EXMC markers show non-specific (or broad) expression in all gastruloid cell types, suggesting that EXMC is a mis-match in gastruloid.
